# Timeless–Tipin interactions with MCM and RPA mediate DNA replication stress response

**DOI:** 10.3389/fcell.2024.1346534

**Published:** 2024-02-29

**Authors:** Paulina Prorok, Eva Wolf, M. Cristina Cardoso

**Affiliations:** ^1^ Cell Biology and Epigenetics, Department of Biology, Technical University of Darmstadt, Darmstadt, Germany; ^2^ Institute of Molecular Physiology (IMP), Johannes Gutenberg-University Mainz, Mainz, Germany

**Keywords:** Timeless-Tipin complex, fork protection complex, DNA replication stress, helicase–polymerase uncoupling, aphidicolin, hydroxyurea, proximity ligation assay

## Abstract

The accuracy of replication is one of the most important mechanisms ensuring the stability of the genome. The fork protection complex prevents premature replisome stalling and/or premature disassembly upon stress. Here, we characterize the Timeless–Tipin complex, a component of the fork protection complex. We used microscopy approaches, including colocalization analysis and proximity ligation assay, to investigate the spatial localization of the complex during ongoing replication in human cells. Taking advantage of the replication stress induction and the ensuing polymerase–helicase uncoupling, we characterized the Timeless–Tipin localization within the replisome. Replication stress was induced using hydroxyurea (HU) and aphidicolin (APH). While HU depletes the substrate for DNA synthesis, APH binds directly inside the catalytic pocket of DNA polymerase and inhibits its activity. Our data revealed that the Timeless–Tipin complex, independent of the stress, remains bound on chromatin upon stress induction and progresses together with the replicative helicase. This is accompanied by the spatial dissociation of the complex from the blocked replication machinery. Additionally, after stress induction, Timeless interaction with RPA, which continuously accumulates on ssDNA, was increased. Taken together, the Timeless–Tipin complex acts as a universal guardian of the mammalian replisome in an unperturbed S-phase progression as well as during replication stress.

## Highlights

- Timeless and Tipin are nuclear proteins present throughout the cell cycle
- The Timeless–Tipin complex is chromatin-bound and replisome-associated in the S-phase
- Upon stress, Timeless association with PCNA drops but interaction with MCM is maintained
- Upon stress, Timeless interaction with RPA increases

## Introduction

Accurate duplication of DNA is one of the major factors in maintaining genome stability. In higher eukaryotes, DNA replication simultaneously starts at multiple sites (origins) within the genome, which imposes a precise replication regulation in time and space. During DNA synthesis, the replication machinery (replisome) can encounter diverse topological, structural, or chemical obstacles that affect the normal progression of the replisome and might induce DNA polymerase stalling. Auxiliary factors, named the fork protection complex (FPC), present within the replisome are responsible for recovery from replication stress and assure continuation of error-free DNA synthesis. The mammalian protein Timeless has been identified as a homolog of the protein TIM from *Drosophila* (Zylka et al., 1998; Takumi et al., 1999) involved in circadian rhythm regulation. In contrast to TIM in *Drosophila,* mammalian Timeless, together with its interaction partner Tipin (Gotter et al., 2007; Yoshizawa-Sugata and Masai, 2007; Grabarczyk, 2020), was shown to be a constitutive part of the replisome, and their depletion results in a decrease in the fork rate even in the absence of genotoxic stress (Gotter et al., 2007; Rageul et al., 2020). An enhancing effect of the Timeless–Tipin complex on mammalian DNA polymerase processivity has been also shown *in vitro* using purified human proteins (Aria et al., 2013; Cho et al., 2013; Baris et al., 2022). Timeless alone has been shown to have a negative effect on the DNA unwinding and ATPase activities of the Cdc45–Mcm2–7–GINS complex in the presence of ssDNA (Cho et al., 2013). Consistent with that, the depletion of Timeless in yeast leads to excessive ssDNA formation, strand breaks, and an increased recombination rate (Sommariva et al., 2005). Under genotoxic stress, Timeless–Tipin-depleted cells fail to activate the replication checkpoint (Unsal-Kaçmaz et al., 2007; Yoshizawa-Sugata and Masai, 2007). Moreover, studies performed in *Caenorhabditis elegans* show an involvement of the Timeless–Tipin complex in replisome disassembly during replication termination (Xia et al., 2021). In humans and *Xenopus laevis*, Timeless–Tipin was also shown to mediate cohesin binding to DNA replication forks and promote the cohesion of newly replicated sister chromatids (Cortone et al., 2018; Errico et al., 2009). However, how the Timeless–Tipin complex fulfills its nuclear functions is unknown. No biochemical activity of the complex has been shown so far. The spatial distribution of the mammalian Timeless–Tipin complex throughout the cell cycle as well as its precise localization within the replisome under normal and replication stress conditions is strongly debated in the literature. Electron microscopy (EM) data evidenced an interaction between mouse Timeless–Tipin and RPA accumulated at ssDNA, but not with ssDNA itself (Witosch et al., 2014). The interaction with other components of the replisome, including polymerases and helicase, is more controversial, and the results obtained using different approaches are not conclusive. A direct interaction of the Timeless–Tipin complex with replicative polymerases has been reported in co-immunoprecipitation experiments in cells (Gotter et al., 2007) and in some (Aria et al., 2013), but not all (Cho et al., 2013), studies of purified human proteins. The above reports led to a model situating the Timeless–Tipin complex between PCNA/DNA polymerase and the replicative helicase (Gotter et al., 2007; Errico et al., 2009; Cho et al., 2013; Witosch et al., 2014). Further studies led to the identification of a DNA/G4-binding domain in human Timeless (Freudenreich, 2020), postulating a new role of Timeless in the detection of obstacles/secondary structures in front of and behind the progressing fork. Timeless depletion in chicken DT40 cells resulted in a lower replication processivity and genetic instability of G4 forming sequences that could be restored by expression of human Timeless (Lerner et al., 2020). Recently, a cryo-EM structure of yeast and human replisomes containing the Timeless–Tipin complex has been resolved, which revealed that the Timeless–Tipin complex is localized in front of the MCM helicase on the dsDNA (Baretić et al., 2020; Jones et al., 2021). This finding led to the postulation of an alternative model in which the Timeless–Tipin complex is situated in front of the MCM helicase on the side of the dsDNA. At this position, the complex could easily detect structural obstacles in DNA prior to their replication and limit MCM helicase activity until the problem is resolved. In contrast, how the Timeless–Tipin complex exerts its enhancing role on the polymerase activities or checkpoint activation becomes even more questionable.

Here, using human cancer cells, the spatial localization of the Timeless–Tipin complex has been addressed. We found that Timeless and Tipin, although abundantly present throughout the cell cycle, are chromatin-bound only during the S-phase. Taking advantage of the naturally occurring helicase–polymerase uncoupling under replication stress conditions, we evaluated the Timeless–Tipin complex’s association with either PCNA/DNA polymerase or the replicative helicase MCM. Upon stress induction, a spatial dissociation between the Timeless–Tipin complex and PCNA/DNA polymerase was observed, but no change was observed in the complex association with MCM helicase. Additionally, we found Timeless–Tipin interactions with RPA upon replication stress induction, evidencing the complex ability to access factors present on ssDNA. Based on our data, the Timeless–Tipin complex acts as a universal guardian of replication fork stability by sensing potential replication problems in front of and behind the moving CMG helicase complex.

## Materials and methods

### Cell culture, replication labeling, and DNA replication stress induction

HeLa Kyoto, HeLa Kyoto mCherry-PCNA, HeLa Kyoto EGFP-RPA2 (Supplementary Table S1), and HEK 293-EBNA cells were cultured in high-glucose Dulbecco’s modified Eagle medium (DMEM) (Cat. No. D6429, Sigma-Aldrich Chemie GmbH, Steinheim, Germany) supplemented with 10% fetal calf serum (Cat. No. F7524, Sigma-Aldrich Chemie GmbH, Steinheim, Germany), 1 × L-glutamine (Cat. No. G7513, Sigma-Aldrich Chemie GmbH, Steinheim, Germany), and 1 μM gentamicin (Cat. No. G1397, Sigma-Aldrich Chemie GmbH, Steinheim, Germany). Prior to the experiment conduction, the cells were seeded on gelatin-coated coverslips. The replication labeling was performed using 5-ethynyl-2′-deoxyuridine (EdU, Cat. No. 7845.1, Carl Roth GmbH) supplementation to the growth medium at a final concentration of 10 µM. Replication labeling was typically performed for 15 min at 37°C. Afterward, the EdU-containing medium was replaced by a medium containing stress inductors. For the control condition (without stress induction), EdU labeling was performed directly prior to cell fixation. DNA replication stress was induced using a medium supplemented with 10 mM hydroxyurea (HU, Cat. No. H8627, Sigma-Aldrich Chemie GmbH, Steinheim, Germany) for 1 h or a medium supplemented with 150 µM aphidicolin (APH, Cat. No. A4487, Sigma-Aldrich Chemie GmbH, Steinheim, Germany) for 30 min. All cells used were tested for *mycoplasma* contamination and used only if not contaminated.

### Cell transfection

HEK 293-EBNA cells were transfected in a 100-mm culture dish using polyethylenimine (PEI) (Cat. No. 40872-7, Sigma-Aldrich Chemie GmbH, Steinheim, Germany). The transfection mixture was obtained using 30 µg of plasmid DNA and 90 µL of 23.3 µM PEI, pH 7.0. First, the plasmid DNA and PEI were mixed separately with 900 µL of DMEM. Subsequently, both mixtures were mixed and incubated at room temperature for 15 min.

The transfection mixture was added dropwise to the dish with cells and incubated overnight before harvesting.

### Western blot analysis

For protein extraction, the cells were resuspended and incubated in RIPA buffer (150 mM NaCl, 1.0% NP-40 (IGEPAL^®^ CA-630), 0.5% sodium deoxycholate, 0.1% SDS, 50 mM Tris, pH 8.0, 5 mM EDTA (pH 8.0), and freshly added 1 mM PMSF) for 30 min at 4°C with end-over-end rotation. The lysates were spun at 13,000 rpm at 4° for 15 min in a benchtop refrigerated centrifuge. The supernatants were harvested, and the pellets were discarded. Next, Laemmli buffer was added, and the samples were boiled at 95° for 5 min before loading them on the gel. The proteins were separated using 10% SDS-PAGE gel and transferred on a 0.2-µm nitrocellulose membrane (BIO-RAD, Cat. No. 1620112). Successful protein transfer was confirmed by Ponceau (Ponceau S solution, Sigma, Cat. No. P7170-1L) staining. After blocking with 3% milk for 30 min at RT, the membrane was incubated overnight with primary antibodies at the dilution specified in Supplementary Table S4. After six washes for 10 min with PBST (1x PBS, Triton 0.1%), incubation with a secondary antibody conjugated with HRP was performed (2 h at RT). Afterward, the membrane was washed three times with PBST, and the signal was revealed using “Clarity Western ECL Substrate” (BIO-RAD, Cat. No. #1705061) and imaged using the Amershan AI600 Imager (GE Healthcare Life Sciences AI600 Imager, Cat. No. 695204). Protein molecular weight ladder used the Color Prestained Protein Standard, Broad Range (10–250 kDa, NEB, Cat. No. P7719S) and PageRuler™ Prestained Protein Ladder (10–180 kDa, Thermo Scientific™, Cat. No. 26616).

### Plasmid

The mammalian expression vectors pEGFP-hTimeless (pc4729) and pEGFP-hTipin (pc4732) were generated by subcloning the human Timeless and human Tipin coding sequences, respectively, into the pENeGFPRPA34 vector (Sporbert et al., 2002). The RPA34 insert was cut out using HindIII (HindIII-HF, NEB, Cat. No. R3104S) and XbaI (XbaI, NEB, Cat. No. R0145S) enzymes. hTimeless and hTipin coding sequences were cloned in frame with GFP using HindIII and XbaI restriction sites. The primers for insert amplification are listed in Supplementary Table S3. The correctness of the sequence was confirmed using sequencing. The primers used for sequencing are listed in Supplementary Table S3.

### Unbound protein pre-extraction and cell fixation

Prior to cell fixation, the unbound protein fraction was pre-extracted using ice-cold 0.5% Triton in 1x PBS for 2 min (for immunostaining with anti-Tipin antibody). Alternatively (for all immunostainings except Tipin), the protein pre-extraction was performed by incubation with a pre-extraction buffer containing Tris-HCl 10 mM, MgCl_2_ 2.5 mM, Nonidet 1%, and PMSF 1 mM for 8 min at 4°C, followed by incubation with washing buffer (Tris-HCl 10 mM, MgCl_2_ 2.5 mM, and PMSF 1 mM) for 5 min at room temperature (RT) with shaking. Cells were fixed using 3.7% formaldehyde (Cat. No. 8775, Sigma-Aldrich Chemie GmbH, Steinheim, Germany) in 1x PBS. Then, the fixed cells were transferred into 1x PBS and stored at 4°C until use.

### Immunostaining, EdU detection, and PLA assay

The fixed cells were permeabilized for 30 min in 1x PBST (0.5% Triton-X 100) at RT, blocked in 2% BSA/1x PBS for 30 min at RT, and immunostained with a selected primary antibody for 1 h at RT, followed by incubation with a corresponding secondary antibody for 45 min at RT. In case of immunostaining using anti-MCM7 and anti-Timeless Alexa Fluor 488-conjugated rabbit antibodies, the cells were incubated with primary rabbit anti-MCM7 antibody first, then with a secondary goat anti-rabbit IgG (H + L) Alexa Fluor 594, and subsequently with rabbit IgG to saturate any binding sites of the secondary antibody and, thus, prevent nonspecific binding of the anti-Timeless Alexa Fluor 488-conjugated rabbit antibody, which was used in the following step of immunostaining. Three washes for 5 min using 1x PBST (0.5% Tween 20) were performed between each antibody incubation step. DNA was counterstained with 4′,6-diamidino-2-phenylindole (DAPI, Cat. No. D27802, Sigma-Aldrich Chemie GmbH, Steinheim, Germany), and coverslips were mounted using Mowiol 4-88 (Cat. No. 81381, Sigma-Aldrich Chemie GmbH, Steinheim, Germany). The EdU incorporated in DNA during replication labeling was detected using Click-iT assay (ROTI^®^Kit for Imaging, Carl Roth) with different dyes (6-FAM, Cat. No. 7773.1, Carl Roth, Karlsruhe, Germany; Eterneon-Red 645 Azide, Cat. No. 1Y73.1, baseclick GmbH, Munich, Germany; and 3-azido-7-hydroxycoumarin dye, Cat. No. BCFA-047-1, baseclick GmbH, Munich, Germany). The antibodies used in immunostaining, PLA assay, and Western blotting with their corresponding dilutions are listed in Supplementary Table S4. The specificity of secondary antibodies was tested by immunostaining without using primary antibodies (according to the scheme in Supplementary Figure S1A). The representative images from this analysis are shown in Supplementary Figure S1B and from colocalization quantification in Supplementary Figure S1C. The PLA assay was performed using Duolink^®^ *in situ* PLA reagents (Duolink^®^ *in Situ* PLA Probe Anti-Rabbit PLUS—DUO92002, Duolink^®^ *in Situ* PLA Probe Anti-Mouse MINUS—DUO92004, and Duolink^®^ *in Situ* Detection Reagents Red, DUO92008, Sigma-Aldrich) according to the manufacturer’s protocol. The specificity of the PLA signal was tested by performing a PLA assay with one primary antibody together with anti-mouse and anti-rabbit PLA probes (scheme in Supplementary Figure S2A). The representative images from this analysis are presented in Supplementary Figure S2B and from signal quantification in Supplementary Figure S2C.

### Microscopy and image processing

Wide-field microscopy imaging was performed using a Nikon CREST Eclipse Ti2 microscope equipped with six solid-state LED light sources (395/25 nm, 440/20 nm, 470/24 nm, 510/25 nm, 550/15 nm, 575/25 nm, and 540/30 nm) using a 40x Plan Apo λ DIC air objective lens and NIS-Elements Advanced Research software. Confocal z-stack imaging was performed using a Leica TCS SPE-II (Wetzlar, Germany) laser scanning confocal microscope equipped with 405 nm, 488 nm, 561 nm, and 633 nm solid-state lasers using an ACS APO 63x/1.3 oil objective lens and Leica LAS X software. Deconvolution of the images was performed using the Iterative Deconvolve 3D ImageJ plugin (https://imagej.net/plugins/iterative-deconvolve-3d). Appropriate theoretical PSF corresponding to the objective and excitation laser used were generated with the Diffraction PSF 3D plugin in ImageJ (https://imagej.net/plugins/diffraction-psf-3d). The colocalization analysis was conducted with the ImageJ Coloc2 plugin (https://imagej.net/plugins/coloc-2).

### Data visualization and statistical data evaluation

RStudio (v1.0.143–v1.1.447) was used for data visualization and statistical evaluation. The data originate from two independent biological replicates. The distribution of collected datasets was evaluated using the Shapiro–Wilk test of normality. For normally distributed datasets, the statistical significance was calculated using a two-tailed unpaired *t*-test between the control and treatment. For other data distributions, the nonparametric Mann–Whitney–Wilcoxon test was performed. Statistical values, including the number of cells analyzed, mean, median, and *p*-values, are summarized in Supplementary Table S5. The statistical significance is indicated as n.s = not significant (*p* > 0.05); * = *p* ≤ 0.05; ** = *p* ≤ 0.01; and *** = *p* ≤ 0.001.

## Results

### Timeless–Tipin complex is chromatin-bound and replisome-associated in the S-phase

The mammalian Timeless–Tipin complex has been described as a part of the replisome and playing a role in DNA replication fork protection. However, there is limited evidence of how Timeless–Tipin fulfills its role during ongoing DNA synthesis and in the replication stress response. To shed light on the human Timeless–Tipin complex spatio-temporal localization in human cells, we characterized both proteins of the complex using immunostaining with antibodies specific for Timeless and Tipin.

**FIGURE 1:**
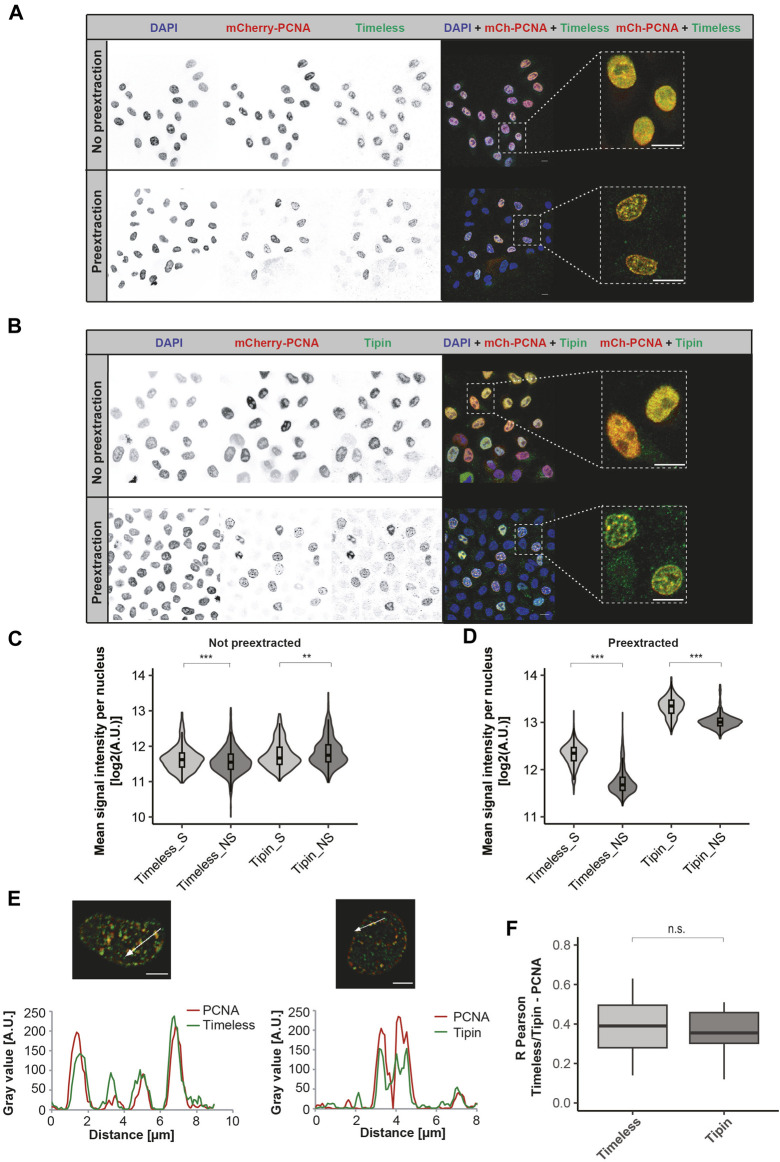
Spatio-temporal localization of the Timeless–Tipin complex in an unperturbed cell cycle. **(A)** Timeless localization in non-pre-extracted fixed cells (top) or after the pre-extraction of chromatin-unbound proteins (bottom). Scale bar 10 µm. **(B)** Tipin localization in non-pre-extracted cells (top) or after chromatin-unbound protein pre-extraction (bottom). Scale bar 10 µm. **(C)** Mean nuclear intensity of Timeless/Tipin signals in non-pre-extracted replicating (S)/not-replicating (NS) cells. N = 694 cells (Timeless S); 1,254 cells (Timeless NS); 304 cells (Tipin S); and 540 cells (Tipin NS). **(D)** Mean nuclear intensity of Timeless/Tipin signals in pre-extracted replicating (S)/not-replicating (NS) cells. N = 1,460 cells (Timeless S); 2,745 cells (Timeless NS); 961 cells (Tipin S); and 768 cells (Tipin NS). **(E)** Fluorescence intensity line profiles of mCherry-PCNA and Timeless signals (left panel) or mCherry-PCNA and Tipin signals (right panel) along the arrow marked in the merged images. The normalized gray value in arbitrary units [A.U.] is plotted as a function of the distance in µm. Scale bar: 5 µm. **(F)** R Pearson correlation of mCherry-PCNA and Timeless or Tipin signals. mCherry-PCNA signals were enhanced by mCherry immunodetection using anti-RFP antibody together with a secondary anti-rat IgG Alexa Fluor 594. The number of observations (N): N (R) = 59 cells (Timeless); 30 cells (Tipin) The statistical significance is calculated as described in Materials and Methods Section and indicated as n.s = not significant (*p* > 0.05).

We first validated the specificity of the antibodies by Western blot analysis of cell extracts from the human HeLa Kyoto cell line (Supplementary Figure S3A, B, left panels) as well as by the detection of recombinant GFP-Timeless and GFP-Tipin after overexpression from a corresponding plasmid in HEK 293-EBNA cells (Supplementary Figure S3A, B, mid and right panels). Immunofluorescence staining of HeLa cells followed by confocal microscopy analysis resulted in the detection of Timeless and Tipin in the cell nuclei in all cells independent of the cell cycle stage (representative images shown in Figure 1A upper panel, 1B upper panel) at similar levels (Figure 1C, statistical significance is related to a high observation number and not to the absolute difference in values observed). A stringent pre-extraction of unbound protein (for details, see Materials and Methods Section) permitted the characterization of the chromatin-bound Timeless–Tipin fraction. To directly relate Timeless–Tipin localization to the position of the replication machinery, we used the HeLa Kyoto cell line stably expressing mCherry-PCNA (Chagin et al., 2016). PCNA is an excellent marker of DNA undergoing replication and exhibits focal patterns in replicating cells that correspond to the position of the replisome (Leonhardt et al., 2000). Immunodetection of Timeless and Tipin in pre-extracted cells showed “replication-like” patterns of both proteins, resembling the patterns of PCNA in replicating cells. Overlap of PCNA and Timeless signals in S-phase cells is visible in Figures 1A, B (lower panels) and magnified on the merged, zoomed image on the right-hand side of both panels. Upon pre-extraction, dramatically lower levels of Timeless/Tipin were observed in non-S-phase cells (Figure 1D), indicating that the chromatin recruitment of the complex is DNA synthesis-related (Figure 1A lower panel, 1B lower panel).

The line profiles traced in arbitrarily selected cells show a similar course of fluorescence intensities for PCNA and Tipin, as well as PCNA and Timeless (Figure 1E). A quantitative evaluation of protein colocalization using the R Pearson correlation coefficient is summarized in Figure 1F and using Manders coefficients M1 and M2 in Supplementary Figures S3B, C, respectively. Overall, similar colocalization values for Timeless–PCNA and Tipin–PCNA are in line with the already reported Timeless–Tipin complex formation. The colocalization analysis was performed using the Coloc2 ImageJ plugin that calculates three parameters: R Pearson measuring the correlation of pixels corresponding to both signals, Manders coefficient M1 measuring the signal overlap colocalization of the first channel (always red, in this case PCNA) with the second one (always green, in this case Timeless or Tipin), and Manders coefficient M2 measuring colocalization of the second channel (Timeless or Tipin) with the first one (PCNA). The detailed colocalization analysis pipeline is presented in Supplementary Figure S4. To improve the quality of the images prior to colocalization measurements, an image deconvolution step was performed. The deconvolution procedure permits reversing the optical light distortion occurring during image acquisition, restoring clearer and sharper images as if the optical distortion did not take place. Deconvolved images were more appropriate for drawing conclusions on the localization properties of the Timeless–Tipin complex. The deconvolution of red and green channels was performed using the Iterative Deconvolution 3D plugin in ImageJ with an appropriate theoretical PFS generated using the Diffraction PSF 3D plugin in ImageJ. To choose the best number of iterations for deconvolution analysis, the outcome of deconvolution in 5, 10, 15, and 20 iterations was evaluated and compared. Deconvolution in five iterations showed the best image improvement parameters, both visual (Supplementary Figure S5A) and when colocalization parameters were measured (Supplementary Figure S5B). Hence, deconvolution in five iterations was applied for all images and analyses presented in this work.

### PCNA and nascent DNA spatially separate from Timeless after fork stalling

**FIGURE 2:**
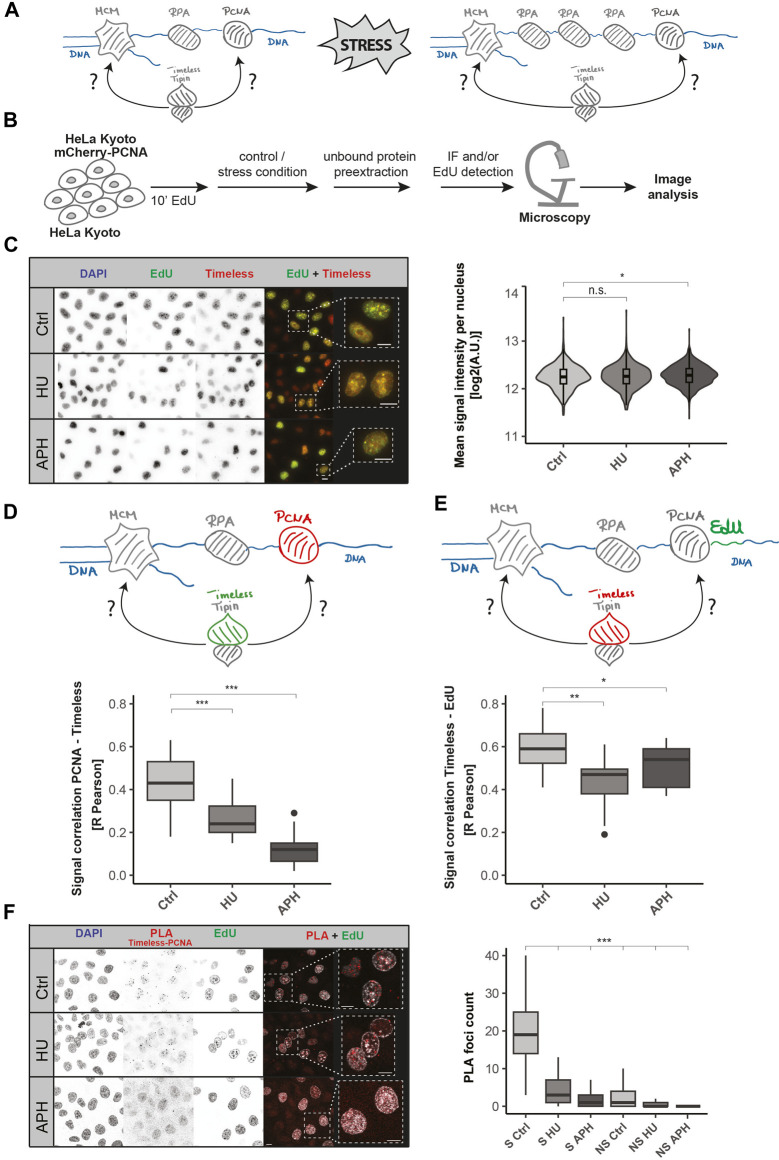
Dynamics of Timeless and PCNA upon replication stress. **(A)** Model of the replisome upon replication stress induction. The Timeless–Tipin complex associates with the replication machinery during DNA synthesis. Replication stress induction leads to PCNA/DNA polymerase dissociation from the MCM helicase complex (polymerase–helicase uncoupling). The position of the Timeless–Tipin complex is addressed in this study. **(B)** Schematic representation of the experimental setup used in this study. HeLa Kyoto cells ectopically expressing mCherry-PCNA were grown in a normal culture medium (control) or in a medium enriched with stress inductors (APH/HU). HeLa Kyoto cells were additionally pulsed with 10 µM EdU for 10 min prior to stress induction. Free nuclear proteins were pre-extracted before cell fixation (for details, see Materials and Methods Section). Subsequently, the immunodetection of Timeless, image acquisition, and analysis were performed. **(C)** Nuclear Timeless signal quantification in replicating cells under normal and stress conditions. Representative images of DNA, EdU, and Timeless signals in control (Ctrl) condition and in replication stress (HU and APH) are shown (left panel). Scale bar: 10 µm. Violin plot of the Timeless’ mean nuclear signal intensity in replicating cells as indicated is shown (right panel). N = 1,109 cells (Ctrl); 945 cells (HU); and 1,475 cells (APH). **(D)** Schematic representation of the association between PCNA (in red) and Timeless (in green) (upper panel). R Pearson correlation of mCherry-PCNA and Timeless signals under the conditions tested (bottom panel). N (R) = 28 cells (Ctrl); 27 cells (HU); and 21 cells (APH). **(E)** Schematic representation of the association between Timeless (in red) and DNA fragments synthesized prior to DNA replication block (EdU signal, in green) (upper panel). R Pearson correlation of mCherry-PCNA and the Timeless signal under the conditions tested (bottom panel). N (R) = 28 cells (Ctrl); 27 cells (HU); and 21 cells (APH). **(F)** Representative images of Timeless–PCNA proximity ligation assay under specified conditions (left panel, scale bar: 10 µm) and PLA signal quantification in S-phase (S) and non-S-phase (NS) cells (right panel). N = 330 cells (S Ctrl); 293 cells (S HU); 315 cells (S APH); 377 cells (NS Ctrl); 413 cells (NS HU); and 757 cells (NS APH). The statistical significance is indicated as n.s = not significant (*p* > 0.05); * = *p* ≤ 0.05; ** = *p* ≤ 0.01; and *** = *p* ≤ 0.001.

To ascertain the precise localization of the Timeless–Tipin complex within the replisome, replication stress induction was performed. The disruption of the DNA synthesis leads to a so-called “helicase–polymerase uncoupling” (Byun et al., 2005; Cortez, 2005; Görisch et al., 2008), the DNA polymerase is stalled, but the DNA helicase continues its action, leading to the generation of long stretches of ssDNA that are coated by RPA, as depicted in Figure 2A. As a consequence, the spatial distance between PCNA/DNA polymerase and the replicative helicase (MCM) increases. Similarly, the stretches of nascent DNA, synthesized before the stress, separate from the MCM complex after the DNA polymerase stalling. In this study, to investigate the localization of the Timeless–Tipin complex within the replisome, we measure the relative colocalization between the Timeless–Tipin complex and other replisome components. A schematic representation of the experiments is depicted in Figure 2B. A 10-min EdU pulse to label nascent DNA prior to the stress was applied, followed by stress induction. Pre-extraction of the unbound nuclear protein fraction was followed by cell fixation and the immunodetection of proteins of interest and/or EdU detection (for details, see Materials and Methods Section). Replication stress was induced using aphidicolin (APH) or hydroxyurea (HU), which differ in the mechanism of action. HU leads to the depletion of dNTPs, whereas APH is a direct inhibitor of the DNA polymerase. As HU is an inhibitor of the ribonucleotide reductase and prevents the synthesis of new dNTPs, in the presence of the drug, the DNA replication continues until the nucleotide pool is used up. In contrast, APH can immediately bind to the DNA polymerase and inhibit the ongoing DNA synthesis. Taking this into account, we conducted the treatment for 30 min and 60 min for APH and HU, respectively.

First, the mean nuclear Timeless signal intensity in replicating cells was investigated. As shown in Figure 2C, Timeless remains bound on the chromatin in the presence of replication stress at unchanged protein levels (representative images, left; quantification, violin plot, right). Timeless nuclear intensity measurement was conducted according to the analysis pipeline shown in Supplementary Figure S6. Next, we analyzed the total nuclear intensities of replication factors investigated in this study in non-pre-extracted cells as well as total EdU intensities upon stress induction. As shown in Supplementary Figure S7A, the levels of PCNA and RPA stay constant when HU stress is applied, and MCM7 shows a mild increase after HU treatment. Upon aphidicolin treatment, a signal increase for all replication factors investigated was observed. In contrast, a minor decrease in the EdU signal (Supplementary Figure S7B) was observed upon stress, which is most likely related to a mild exonuclease activity at reversed forks. Subsequently, the chromatin-bound levels of PCNA and RPA were investigated (Supplementary Figure S7B). In concordance with previously published data (Görisch et al., 2008), the induction of stress leads to a decreased PCNA level and increased RPA level on the chromatin (Supplementary Figure S7C).

Next, the colocalization between Timeless and PCNA was examined (Figure 2D), and the representative images are shown in Supplementary Figure S8A (left panel). A successful stress induction was tested using an EdU pulse after the HU/APH treatment, as depicted in Supplementary Figure S8B. The presence of the EdU signal in the control (Ctrl) and its lack in HU and APH conditions confirm a successful DNA synthesis disruption (Supplementary Figure S8C, left panel). Both HU and APH led to a significant drop in Timeless and mCherry-PCNA colocalization when based on the R Pearson coefficient (Figure 2D) as well as both Manders coefficients, M1 and M2 (Supplementary Figure S8D). The previously reported dissociation of PCNA upon DNA polymerase inhibition (Görisch et al., 2008) might have contributed to the decrease in colocalization. To further test this, we evaluated the colocalization between Timeless and DNA fragments synthesized prior to the stress induction. The nascent DNA, in contrast to PCNA, is not affected by binding-dissociation dynamics and can be used as a reliable colocalization measurement. In order to mark the nascent DNA, we applied a short EdU pulse before the stress induction and subsequently performed Timeless and EdU detection (Supplementary Figure S8B, right panel). Like the previous experiment, the success of the treatment was confirmed using an EdU pulse after the stress induction and according to the scheme in Supplementary Figure S8B (Supplementary Figure S8C, right panel). Similar to PCNA, EdU signals colocalized very well with Timeless in unperturbed DNA replication. DNA replication disruption led to a drop in the colocalization (R Pearson coefficient, Figure 2E, representative images shown in Supplementary Figure S8A, right panel), which is in line with the stress-related decrease in colocalization between PCNA and Timeless (Figure 2D). The same tendencies in colocalization were also observed when using Manders coefficients M1 and M2 (Supplementary Figure S8E).

Finally, we further tested Timeless and PCNA spatial proximity using the proximity ligation assay (PLA). PLA allows the *in situ* detection of the spatial proximity of two protein targets detected with the help of two primary antibodies raised in different species. We used an anti-PCNA antibody raised in mice and an anti-Timeless antibody raised in rabbits. The PLA assay was evaluated as shown in Supplementary Figure S9. A substantial number of Timeless–PCNA PLA foci were observed under normal conditions in replicating cells with the non-S-Phase cells being free of PLA signals. Significantly fewer PLA foci were detected when HU or APH treatment was applied (Figure 2F). Hence, the outcome of the PLA assay corroborates our previous observation from the colocalization analysis.

### Colocalization of Timeless and MCM7 is not affected by DNA replication stress

**FIGURE 3:**
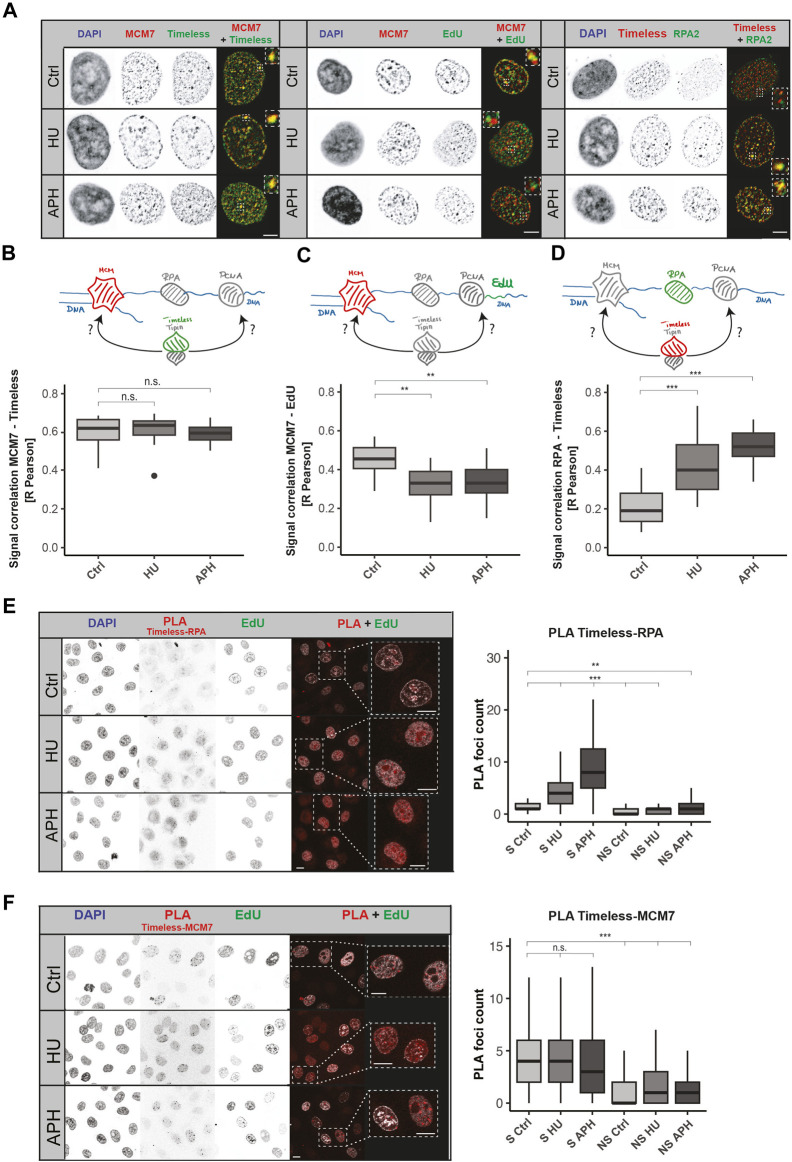
Both MCM7 and RPA interact with Timeless upon replication stress. **(A)** Representative images of Timeless, MCM7, nascent DNA fragments, and RPA nuclear localization in normal conditions and upon replication stress, as indicated. Scale bar: 5 µm. **(B)** Schematic representation of the association between MCM7 (in red) and Timeless (in green) (upper panel). R Pearson correlation of MCM7 and Timeless signals under the conditions tested (bottom panel) is shown. N (R) = 32 cells (Ctrl); 24 cells (HU); and 23 cells (APH). **(C)** Schematic representation of the association between MCM7 (in red) and nascent DNA fragments, EdU (in green) (upper panel), and the respective R Pearson correlation coefficient (bottom panel). N (R) = 22 cells (Ctrl); 21 cells (HU); and 25 cells (APH). **(D)** Schematic representation of the association between Timeless (in red) and RPA (in green) (upper panel) and the respective R Pearson correlation coefficient (bottom panel). N (R) = 31 cells (Ctrl); 46 cells (HU); and 25 cells (APH). **(E)** Representative images of Timeless–RPA proximity ligation assay as indicated are shown (left panel). Scale bar: 10 µm. PLA signal quantification in S-phase (S) and non-S-phase (NS) cells (right panel) is shown. N = 327 cells (S Ctrl); 211 cells (S HU); 355 cells (S APH); 325 cells (NS Ctrl); 132 cells (NS HU); and 201 cells (NS APH). **(F)** Representative images of Timeless–MCM7 proximity ligation assay as indicated (left panel). Scale bar: 10 µm. PLA signal quantification in S-phase (S) and non-S-phase (NS) cells is shown (right panel). N = 229 cells (S Ctrl); 272 cells (S HU); 311 cells (S APH); 331 cells (NS Ctrl); 341 cells (NS HU); and 318 cells (NS APH). The statistical significance is indicated as n.s = not significant (*p* > 0.05); * = *p* ≤ 0.05; ** = *p* ≤ 0.01; and *** = *p* ≤ 0.001.

Next, we needed to dissect the relative position of Timeless with other replisome components, including the DNA helicase. It is particularly interesting because of the polymerase–helicase uncoupling upon replication stress. The DNA helicase does not sense the disruption of replication progression and continues unwinding the DNA double helix (Lopes et al., 2006; Taylor and Yeeles, 2018; Görisch et al., 2008). As a consequence, long ssDNA stretches are formed, and proteins associated with MCMs spatially separate from the replicative polymerase. To draw conclusions of Timeless association either with PCNA/DNA polymerase or replicative helicase, we measured the relative localization between Timeless and MCM7 under normal and stress conditions. The representative images from MCM7 and Timeless immunodetection are shown in Figure 3A (left panel). The successful stress induction, similarly to the previous experiments, was examined using an EdU pulse after treatment according to the scheme in Supplementary Figure S8B and shown in Supplementary Figure S10A. MCM7 and Timeless showed a high colocalization in an unperturbed S-phase that was not influenced by replication stress when the R Pearson correlation coefficient (Figure 3B) or Manders M1 and M2 colocalization coefficients were used (Supplementary Figure S10B). These results suggest that MCM7 and Timeless remain in spatial proximity when replication stress is applied.

To test the spatial separation between MCM and the stalled DNA polymerase, we examined the colocalization between MCM7 and nascent DNA fragments synthesized before DNA replication disruption (representative images in Figure 3A, middle panel). A successful stress induction was tested as in previous experiments, and the results are represented in Supplementary Figure S10A (right panel). The colocalization measurement showed a drop of colocalization between MCM7 and EdU (Figure 3B; Supplementary Figure S10C), in line with the “polymerase–helicase uncoupling.”

Based on these results, we could conclude that in an unperturbed S-phase, the Timeless–Tipin complex moves together with other replisome components, but when the replication is disrupted, Timeless stays associated with the MCM helicase and spatially separates from the stalled PCNA/DNA polymerases. The question whether the complex can access factors situated on the ssDNA side remains open. The Timeless–Tipin complex’s role in DNA damage signaling and replication checkpoint induction most likely requires interaction with stress response factors, as indicated by the previously reported complex interaction with RPA (Witosch et al., 2014). Hence, we next evaluated the colocalization between Timeless and RPA. RPA protein protects ssDNA formed during the normal progression of the replication fork but is particularly enriched in replication stress conditions. An accumulation of RPA in the vicinity of the stalled replication fork is one of the replication stress hallmarks. For an easier evaluation of RPA accumulation upon stress, we used a HeLa cell line ectopically expressing GFP-RPA (Pabba et al., 2023). Figure 3A (right panel) shows a large accumulation of RPA after HU/APH treatment, which confirms a successful stress induction. Additionally, the lack of EdU incorporation in treated cells was tested and is shown in Supplementary Figure S10D. After both APH and HU treatments, an increase in Timeless–RPA colocalization was observed for all parameters measured (R Pearson, Figure 3D; M1 and M2 Manders, Supplementary Figure S10E), indicating that Timeless localizes in the vicinity of ssDNA stretches formed upon replication stress. To obtain a further line of evidence, we performed a PLA assay between Timeless and RPA as well as Timeless and MCM7. The PLA assay permitted achieving a better resolution in protein–protein spatial proximity assessment as the colocalization measurement. The Timeless–RPA PLA foci formation was strongly increased when the replication stress was applied (Figure 3E), whereas the Timeless–MCM7 PLA showed constant PLA signal levels in both the unperturbed S-phase and in replication stress (Figure 3F).

## Discussion

**FIGURE 4:**
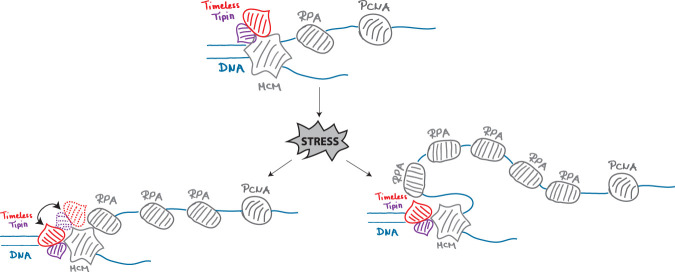
Model for the localization of the human Timeless–Tipin complex within the replisome under normal and replication stress conditions.

The Timeless–Tipin complex is a component of the mammalian replisome, a member of the FPC, but how it fulfills its role in fork protection is unclear. Early studies revealed a positive effect of Timeless–Tipin on replication efficiency and a negative effect on the replicative helicase (Cho et al., 2013) as well as complex interaction with RPA. Consistent with these results, a model situating Timeless–Tipin between DNA polymerase and helicase has been proposed. The cryo-EM structure of the yeast and human replisome situated the Timeless–Tipin complex before the CMG helicase (Baretić et al., 2020; Jones et al., 2021). At this position, the complex can easily detect structural obstacles in DNA prior to their replication and limit MCM helicase activity until the problem is resolved. However, its role in replication activity and reversed fork protection upon stress is much more challenging to imagine. To address these controversies, here we present a comprehensive characterization of the spatial localization of the human Timeless–Tipin complex in an unperturbed S-phase as well as upon stress. Moreover, using the proximity ligation assay, we tested how the interactions of the proteins in the replisome evolve when DNA replication is challenged. We evidenced that the Timeless–Tipin complex colocalizes with the replisome components during ongoing DNA replication progression (Figures 1E, F) and stays bound on the chromatin at unchanged levels even upon a prolonged replication fork stalling (Figure 2C). However, the Timeless–PCNA interaction is lost when replication stress is applied (Figure 2F) but not the interaction with the MCM helicase (Figure 3F), which is in line with the complex replisome position according to the cryo-EM structure of the human replisome. Interestingly, we observed an increased colocalization between Timeless and RPA (Figure 3D) as well as increased interactions between these proteins in PLA assay under replication stress conditions (Figure 3E). These results corroborate the previously evidenced Timeless–Tipin–RPA interactions using electron microscopy approaches (Witosch et al., 2014) and cell-based assays (Kemp et al., 2010). The ability of Timeless–Tipin to interact with RPA accumulated at ssDNA points out the necessity of the complex to directly sense replication problems and transmit the signal of DNA replication stress, leading to the DNA replication checkpoint activation. How the dsDNA-bound Timeless–Tipin complex accesses the ssDNA-bound RPA has to be clarified. Recruitment of new Timeless–Tipin molecules through RPA seems not to be plausible, as the observed Timeless–Tipin levels before and after stress induction remain the same (Figure 2C). RPA accumulation in the unperturbed S-phase mostly occurs at the lagging strand and after stress induction at both lagging and leading strands. Through the flexible structure of RPA-loaded ssDNA, ssDNA–RPA could bend and become proximal to the Timeless–Tipin complex. This could be a model of Timeless–RPA interaction upon stress. Another possible explanation would be a remodeling of the replisome architecture, including the transfer of at least some Timeless–Tipin molecules to the ssDNA side coated with RPA (see the model in Figure 4). Another possible explanation would be a remodeling of the replisome architecture, including the transfer of at least some Timeless–Tipin molecules to the ssDNA side. As the Timeless–RPA association was revealed to be the most pronounced upon APH treatment in both colocalization analysis (Figure 3D) and PLA assay (Figure 3E), it is possible that remodeling is dependent on the stress inductor. Remodeling of the Timeless position within the replisome has been already proposed in the literature. Somyajit et al. reported the loss of Timeless from the nascent DNA upon HU treatment at concentration and time comparable to those used in this study (Somyajit et al., 2017). As the authors analyzed the nascent DNA, the apparent loss of Timeless can be associated with its spatial dissociation from nascent DNA, which is also observed in our study (Figure 3C). Moreover, upon Timeless knockdown together with HU treatment, an excessive MRE11-dependent degradation of reversed forks has been reported, pointing to an active role at the stalled/reversed fork. Interestingly, the same study reports Timeless retention on the chromatin when the stress is APH-induced, which can be associated with a different mechanism of action of both compounds. APH treatment may reduce the MCM helicase rate and limit the spatial dissociation between Timeless and nascent chromatin that was also observed in our study (Figure 2E). In light of the current knowledge, it is, however, much more challenging to explain how the DNA polymerase activity stimulation by Timeless occurs. An indirect signal transmission through interaction with other FPC members seems to be the most plausible explanation. Timeless–Tipin was shown to interact with CLASPIN (Gotter et al., 2007; Jones et al., 2021; Baris et al., 2022), another FPC member. Depletion of CLASPIN weakens the enhancing effect of Timeless–Tipin on replicative polymerase, and all three proteins are important to achieve a normal fork rate (Gotter et al., 2007; Baris et al., 2022). SDE2 is another factor integrating the Timeless–Tipin complex in replication fork stability (Rageul et al., 2020). Depletion of SDE2 or Timeless results in slowed-down fork progression, defects in stalled fork recovery, checkpoint activation failure, and the degradation of reversed forks (Rageul et al., 2020). As per the present knowledge, Timeless–Tipin functions rely on the complex interaction with other replisome components and crosstalk between them. In Figure 4, we propose a model of Timeless–Tipin localization within the replisome. In an unperturbed S-phase, Timeless–Tipin locates ahead of the MCM helicase, sensing hindrances for replisome progression. When replication stress is induced, Timeless–Tipin can access stress response factors accumulated on ssDNA in the ways depicted, thus permitting an efficient stress signaling response.

## Data Availability

The original contributions presented in the study are included in the article/Supplementary Material, further inquiries can be directed to the corresponding author. All data is available from: https://doi.org/10.48328/tudatalib-1282.
