# Supplementary material for: Timeless–Tipin interactions with MCM and RPA mediate DNA replication stress response
Source: Front Cell Dev Biol. 2024 Feb 29;12:1346534. doi: 10.3389/fcell.2024.1346534 (PMC10939015; doi:10.3389/fcell.2024.1346534)
Supplement: Supplementary file 1 [file Presentation1.pdf]

SUPPLEMENTARY MATERIAL

Timeless-Tipin interactions with MCM and RPA mediate DNA replication stress response

Paulina Prorok[1], Eva Wolf[2] and M. Cristina Cardoso[1]

[1]Cell Biology and Epigenetics, Department of Biology, Technical University of Darmstadt, 64287 Darmstadt, Germany.

[2]Institute of Molecular Physiology (IMP), Johannes Gutenberg-University Mainz, 55128 Mainz, Germany.

SUPPLEMENTARY FIGURES

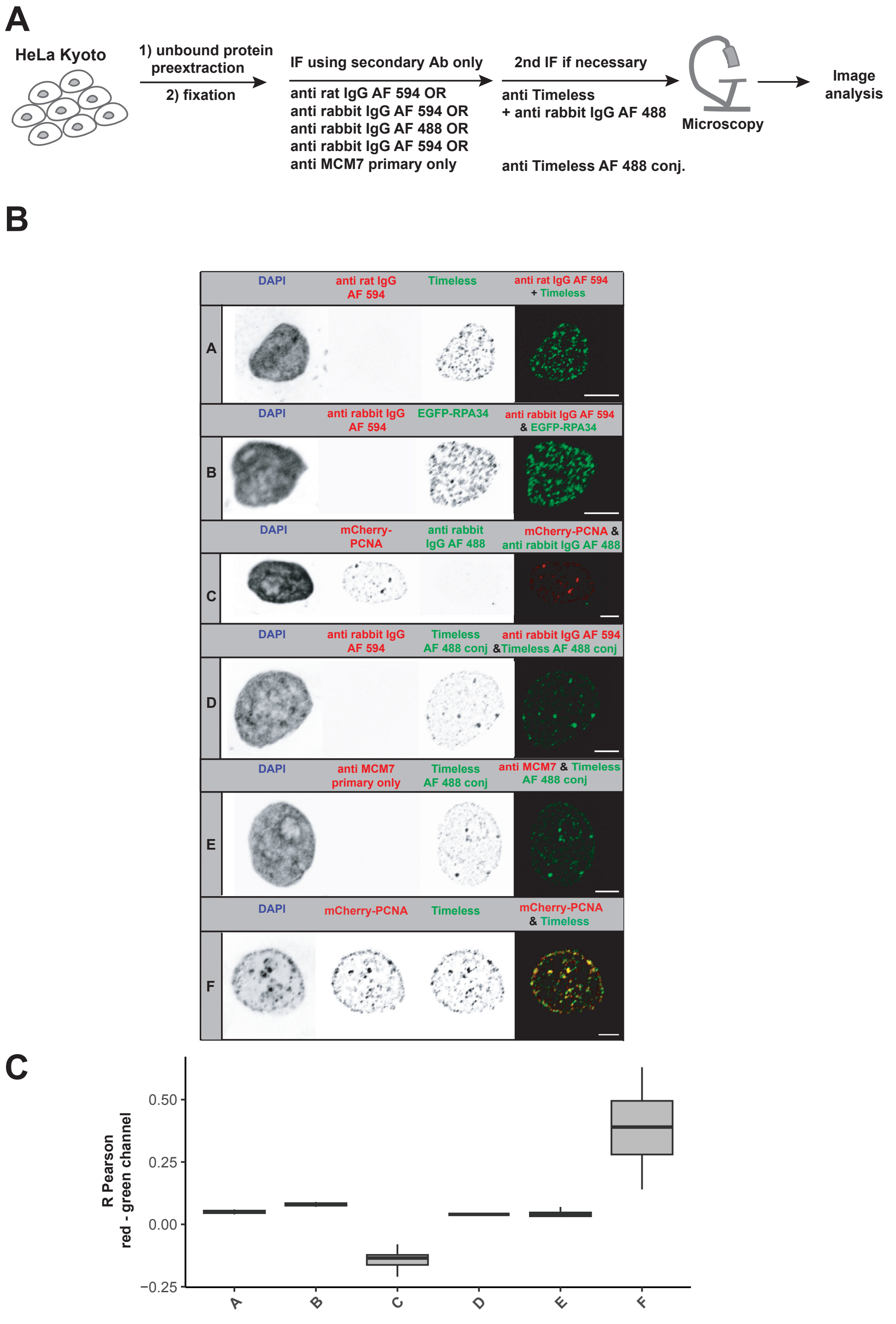

Figure S1. Secondary antibody specificity.

(A) Schematic representation of the experimental setup used to test the specificity of secondary antibodies used in the study. **(B)** Representative images of immunodetection of proteins of interest using indicated antibodies. Selected secondary or primary antibodies were used to evaluate their non-specific binding and background noise they generate. Scale bar: 5 µm. **(C)** R Pearson correlation measurement between red and green channels corresponding to experimental conditions shown in A and B. For the comparison purposes the R Pearson correlation between mCherry-PCNA and Timeless was plotted. N (R) = 2 cells (A); 2 cells (B); 6 cells (C); 1 cell (D); 6 cells (E); 59 cells (F).

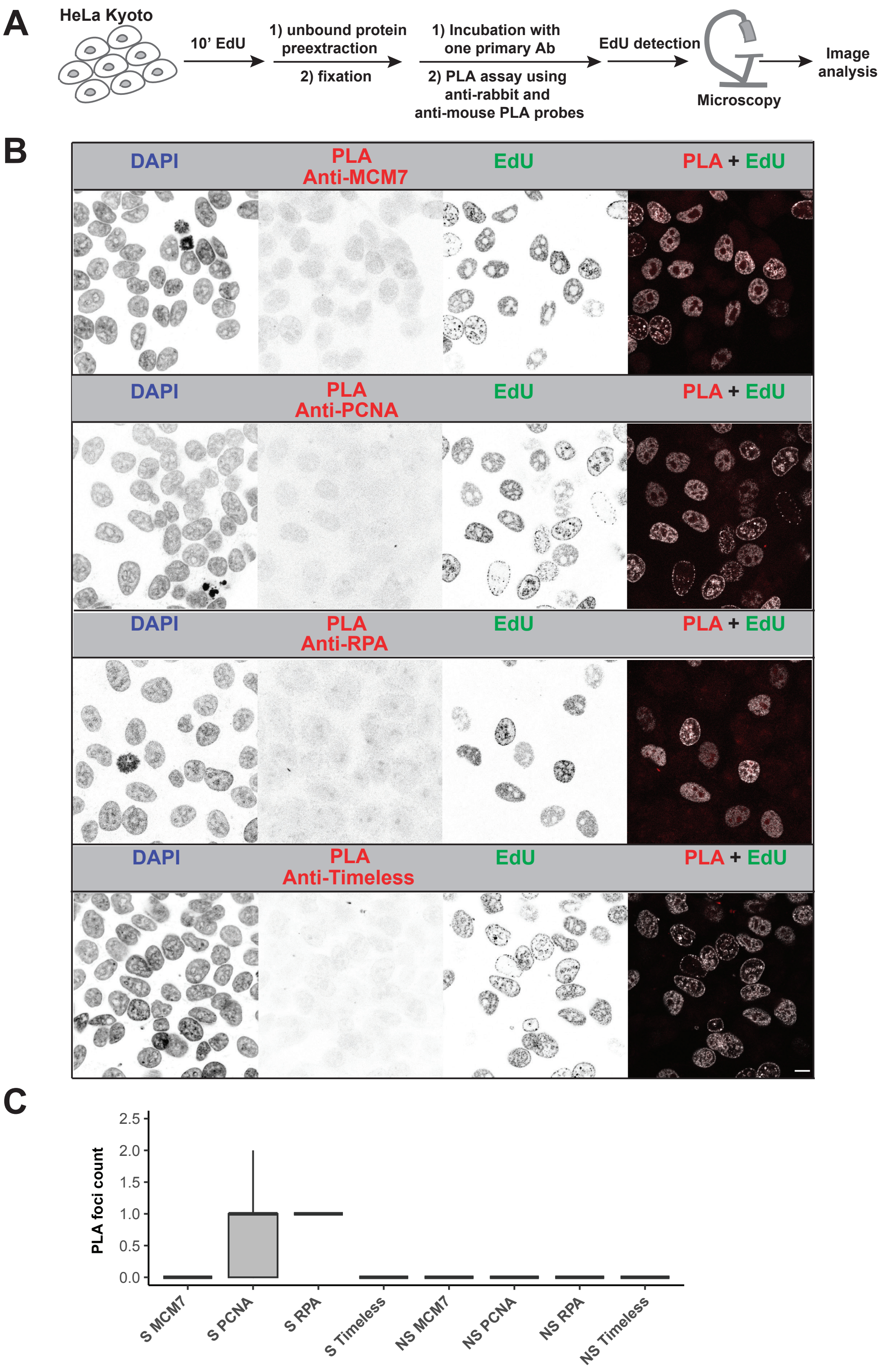

Figure S2. PLA experiment controls.

(A) Schematic representation of the experimental setup used to test the specificity of PLA signals. In order to verify if PLA signal is not generated by a single primary antibody, a PLA assay using one primary antibody and both PLA probes was performed. **(B)** Representative images of PLA assay using indicated antibodies. Scale bar: 10 µm. **(C)** PLA foci count in S-phase (S) and non S-phase (NS) cells using indicated antibodies. N = 105 cells (S MCM7); 95 cells (S PCNA); 139 cells (S RPA); 88 cells (S Timeless); 293 cells (NS MCM7); 223 cells (NS PCNA); 193 cells (NS RPA); 215 cells (NS Timeless).

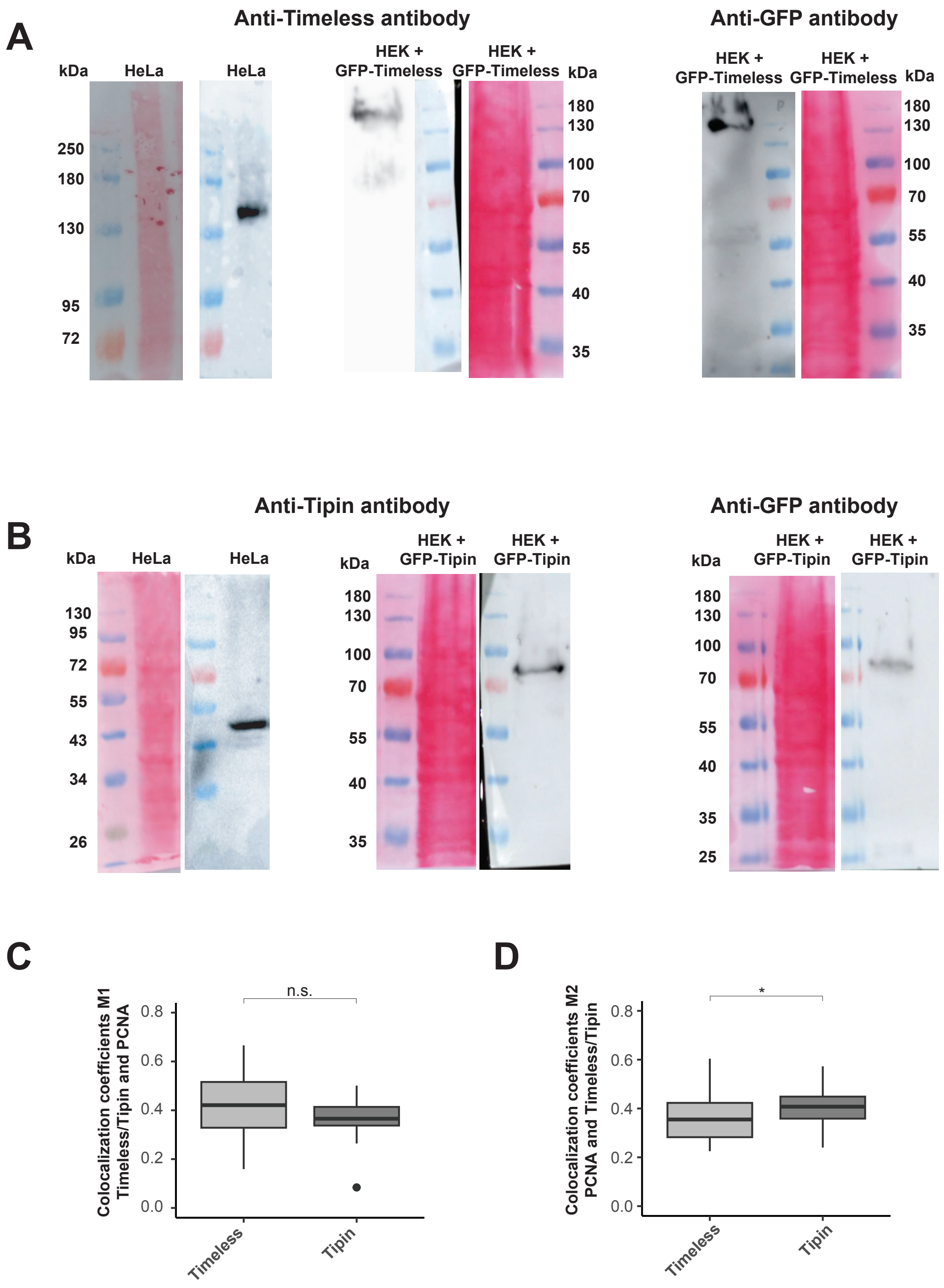

Figure S3. Timeless and Tipin colocalize with the replisome in S-phase cells.

(A) Timeless detection in HeLa cell extracts using Western blot analysis (left panel). The successful transfer of proteins onto the membrane was confirmed by Ponceau staining (left panel). Immunodetection signal is shown in the right panel as indicated. Middle and right panels represent detection of GFP-Timeless after overexpression in HEK-EBNA cells using anti-Timeless (middle) and anti-GFP (right) antibodies. **(B)** Tipin detection was performed as in (A). **(C)** Manders coefficient M1 indicating colocalization of Timeless/Tipin signals with PCNA. N (M1) = 59 cells (Timeless); 30 cells (Tipin). **(D)** Manders coefficient M2 indicating colocalization of PCNA signal with Timeless/Tipin. N (M2) = 59 cells (Timeless); 30 cells (Tipin). The statistical significance (for details see Method section) is indicated as: n.s. = not significant ($p > 0.05$); * = $p \leq 0.05$.

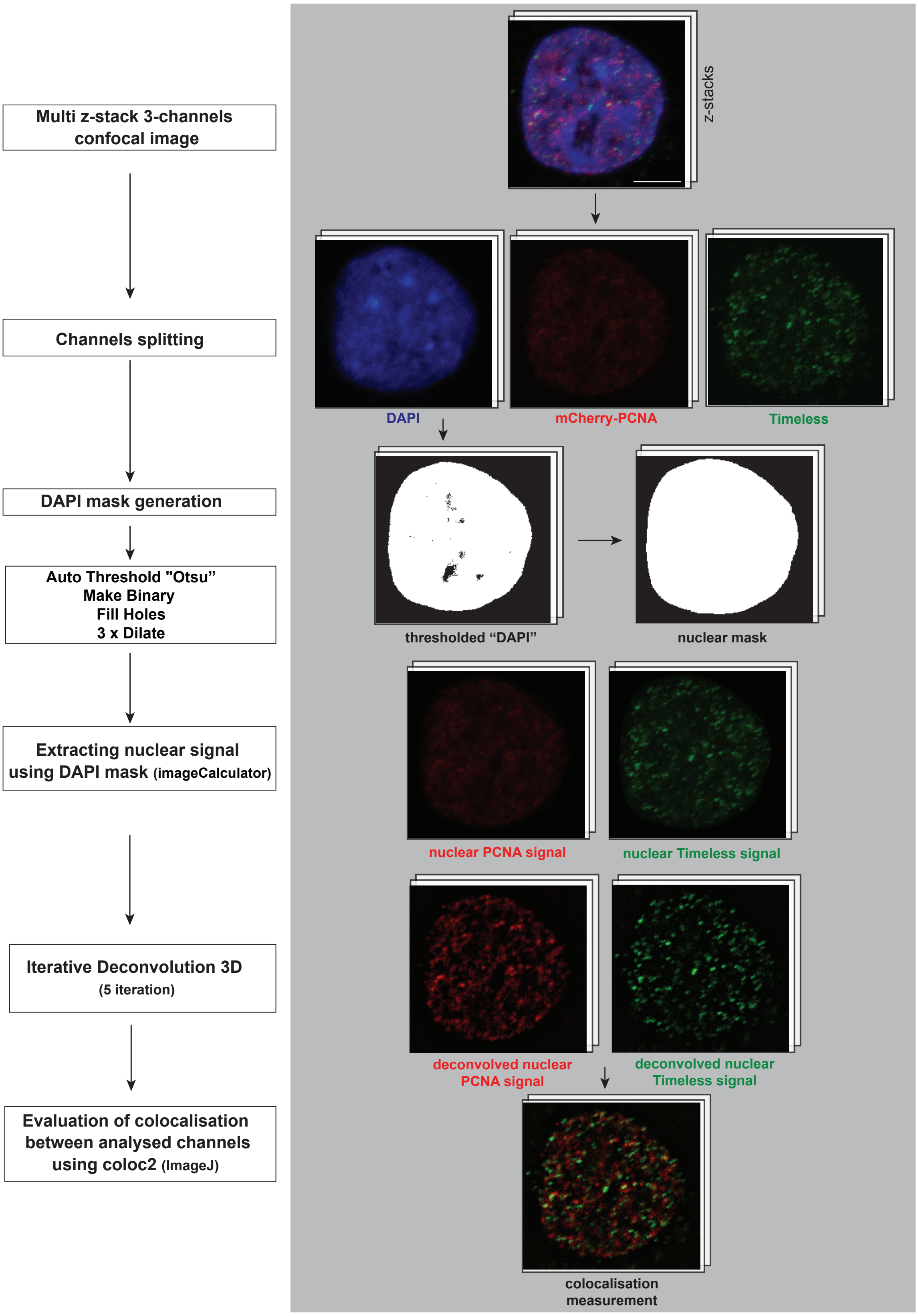

Figure S4. Pipeline for colocalization analysis.

Confocal z-stack images were split into separate channels. DAPI (blue) channel was then used for nuclear-mask generation. The mask was used for nuclear signal extraction from red and green channels. After subsequent deconvolution of images, performed in 5 iterations using Iterative Deconvolution 3D ImageJ plugin, the colocalization analysis was performed using coloc2 plugin in ImageJ. Scale bar 5 µm.

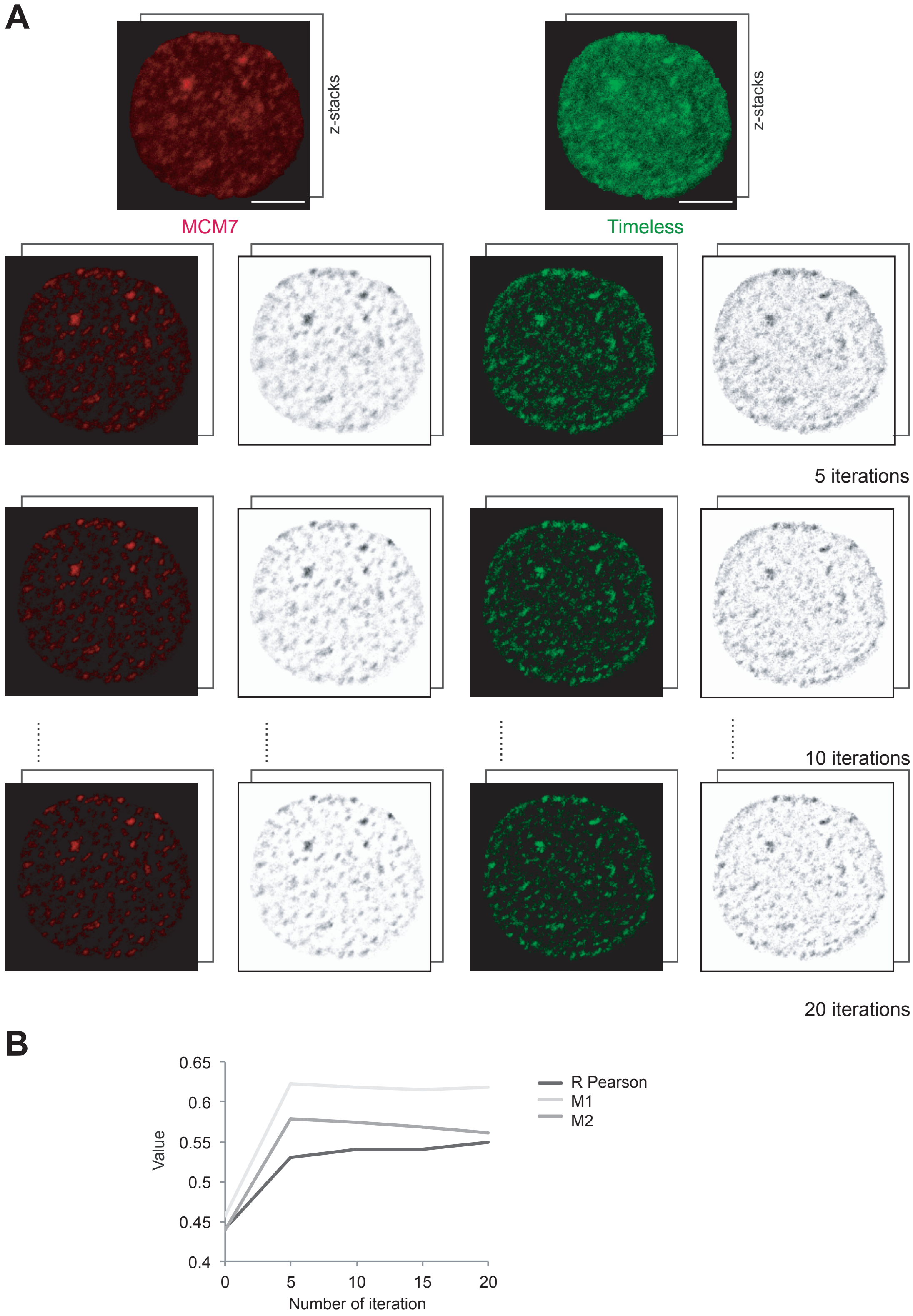

Figure S5. Deconvolution analysis.

(A) To test the effect of deconvolution on image quality, selected images were deconvolved over 5, 10, 15 and 20 iterations as indicated. Scale bar 5 µm. **(B)** Deconvolved images were subsequently used in the colocalization analysis with coloc2 plugin in ImageJ. The graph represents obtained results.

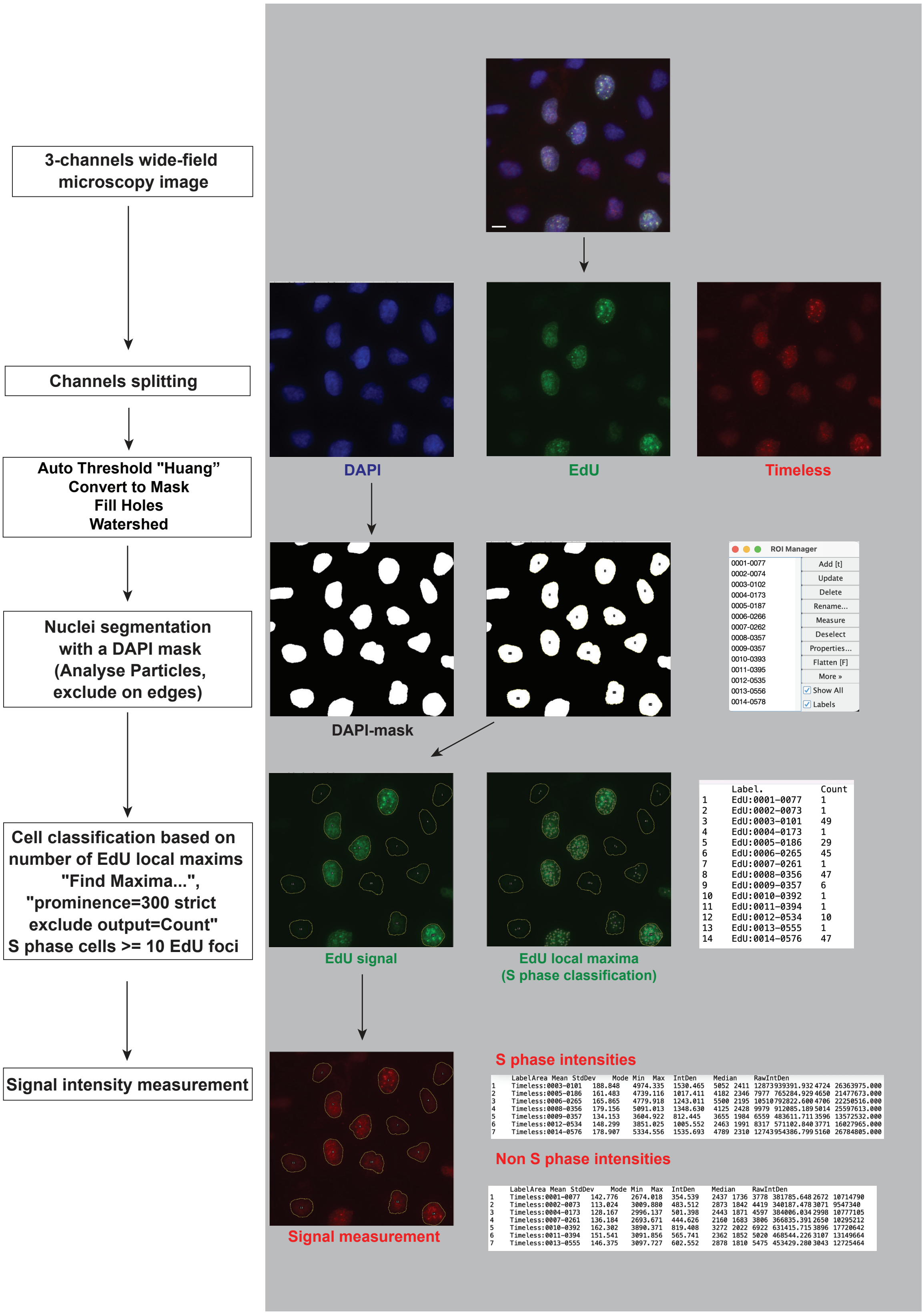

Figure S6. Pipeline for nuclear intensity signal measurement.

Multichannel wide-field images were splitted into separate channels. DAPI (blue) channel was then used for nuclear-mask generation. The cells were classified into S and non S-phase cells based on the number of local maxima in the EdU channel. Nuclei with a count of more than 10 local maxima indicated an S-phase cell. Subsequently, the nuclear signal intensity of the proteins was measured. Scale bar 10 μm.

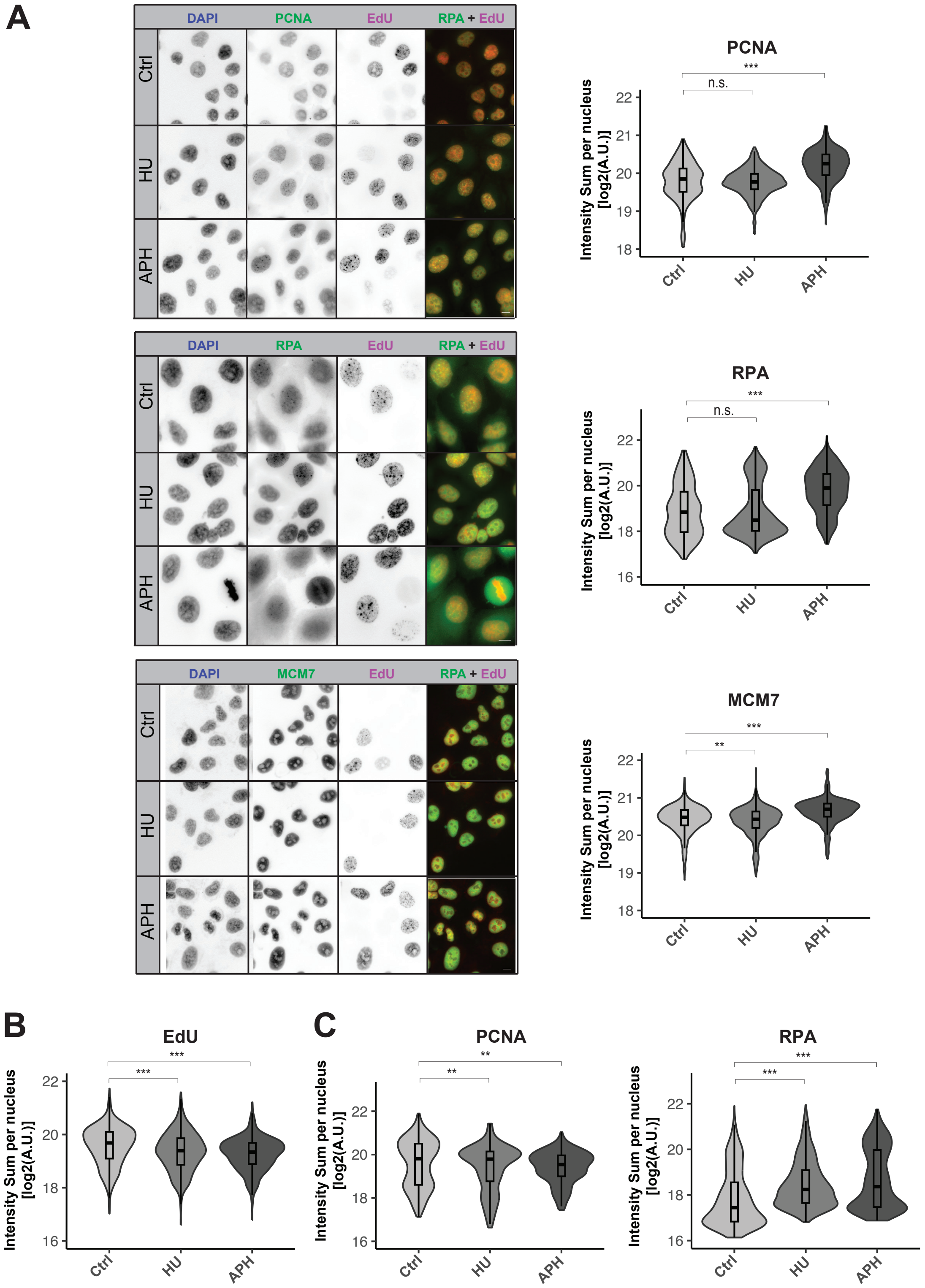

Figure S7. Replication factor levels in unperturbed S-phase and upon replication stress.

(A) Representative images of detection of EdU, PCNA, RPA and MCM7 in not preextracted HeLa Kyoto cells under the conditions indicated. (left panel). Scale bar: 10 µm. Violin plots (right panel) represent the quantification of the total nuclear intensity of the corresponding signals. N: (PCNA) = 250 cells (Ctrl); 578 cells (HU); 396 cells (APH). N: (RPA) = 380 cells (Ctrl); 996 cells (HU); 667 cells (APH). N (MCM7) = 783 cells (Ctrl); 705 cells (HU); 371 cells (APH). (B) Nuclear intensity of total EdU signal in normal condition and after HU/APH-mediated replication stress. N (EdU) = 1109 cells (Ctrl); 945 cells (HU); 1475 cells (APH). (C) Total intensity of chromatin-bound fraction of PCNA (left panel) and RPA (right panel) under the conditions indicated. N (PCNA) = 539 cells (Ctrl); 295 cells (HU); 254 cells (APH). N (RPA) = 847 cells (Ctrl); 1383 cells (HU); 751 cells (APH). The statistical significance is indicated as: n.s. = not significant ($p > 0.05$); * = $p \leq 0.05$; ** = $p \leq 0.01$; *** = $p \leq 0.001$.

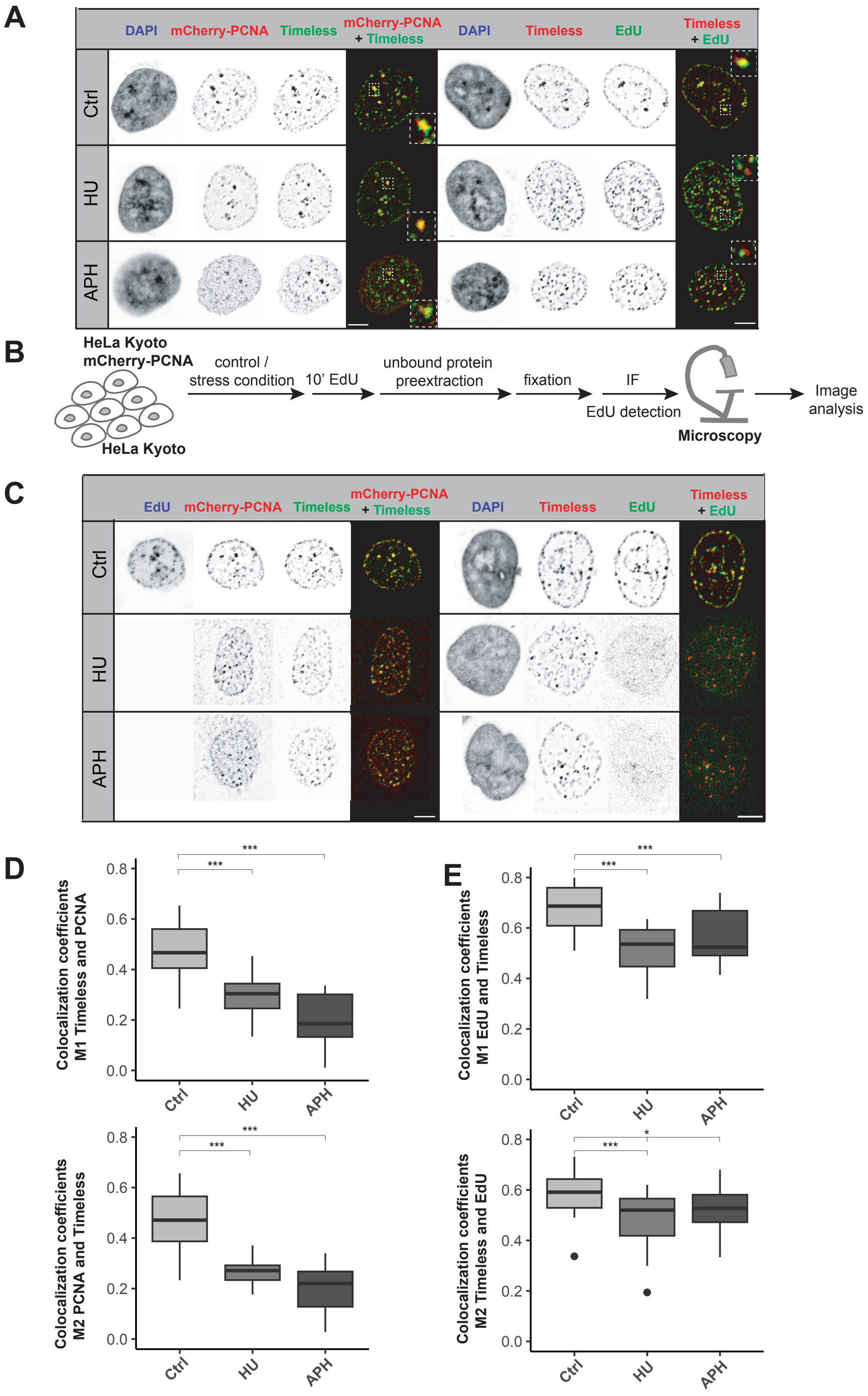
A
DAPI
mCherry-PCNA
Timeless
mCherry-PCNA + Timeless
DAPI
Timeless
EdU
Timeless + EdU
Ctrl
HU
APH
B
HeLa Kyoto mCherry-PCNA
HeLa Kyoto
control / stress condition
10' EdU
unbound protein preextraction
fixation
IF
EdU detection
Microscopy
Image analysis
C
EdU
mCherry-PCNA
Timeless
mCherry-PCNA + Timeless
DAPI
Timeless
EdU
Timeless + EdU
Ctrl
HU
APH
D
Colocalization coefficients M1 Timeless and PCNA
Colocalization coefficients M2 PCNA and Timeless
E
Colocalization coefficients M1 EdU and Timeless
Colocalization coefficients M2 Timeless and EdU
Ctrl
HU
APH
***
*

Figure S8. Replication stress induces dissociation of Timeless from PCNA.

(A) Representative images of detection of Timeless in HeLa Kyoto cells stably expressing mCherry-PCNA (left panel) or Timeless and EdU in HeLa Kyoto (right panel) cells under indicated condition. Scale bar: 5 µm. **(B)** Schematic representation of the experimental setup used to validate the stress induction. HeLa Kyoto cells were cultivated in normal culture medium (Ctrl condition), in medium containing 10 mM HU for 1 h or 150 µM APH for 30 min. After treatment, a 10 µM EdU pulse for 10 min was applied, followed by chromatin-unbound protein preextraction and cell fixation. Subsequently, the detection of Timeless and EdU was performed. **(C)** Representative images of detection of Timeless and EdU in HeLa Kyoto cells stably expressing mCherry-PCNA (left panel) or HeLa Kyoto (right panel) as indicated. Scale bar: 5 µm. **(D)** Manders coefficient M1 indicating colocalization of Timeless and mCherry-PCNA signals (upper panel). Manders coefficient M2 indicating colocalization of mCherry-PCNA and EdU signals (lower panel). N (M1, M2) = 25 cells (Ctrl); 22 cells (HU); 24 cells (APH). **(E)** Manders coefficient M1 indicating colocalization of EdU and Timeless signals (upper panel). Manders coefficient M2 indicating colocalization of Timeless and EdU signals (lower panel). N (M1, M2) = 28 cells (Ctrl); 27 cells (HU); 21 cells (APH). The statistical significance is indicated as: n.s. = not significant ($p > 0.05$); * = $p \leq 0.05$; ** = $p \leq 0.01$; *** = $p \leq 0.001$.

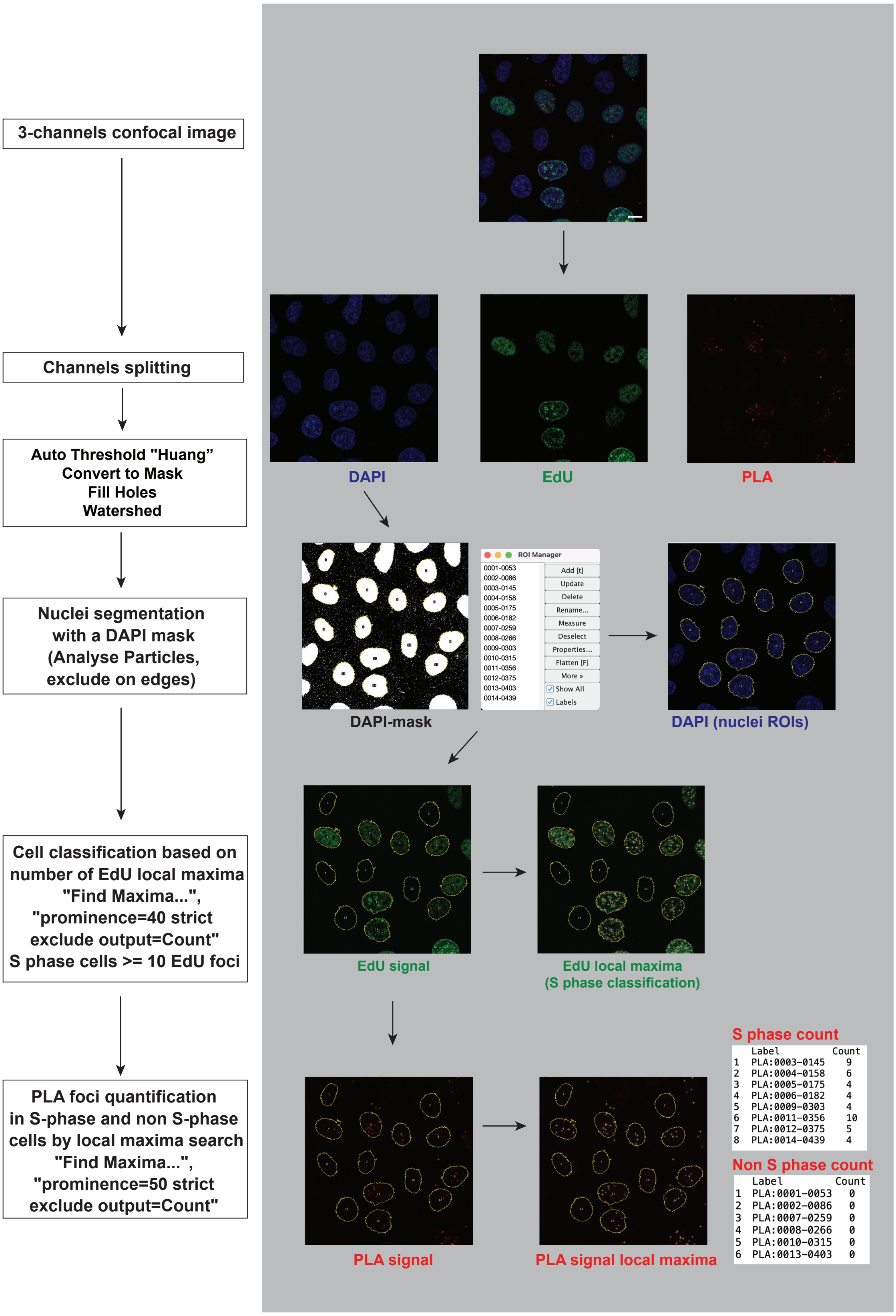

Figure S9. Pipeline for PLA signal evaluation.

Multichannel confocal images were splitted into separate channels. DAPI (blue) channel was then used for nuclear-mask generation. The cells were classified into S and non S-phase cells based on the EdU foci number (S phase >10 local maxima) . Subsequently, the number of PLA foci was counted in both S and non S-phase cells. Scale bar 10 µm.

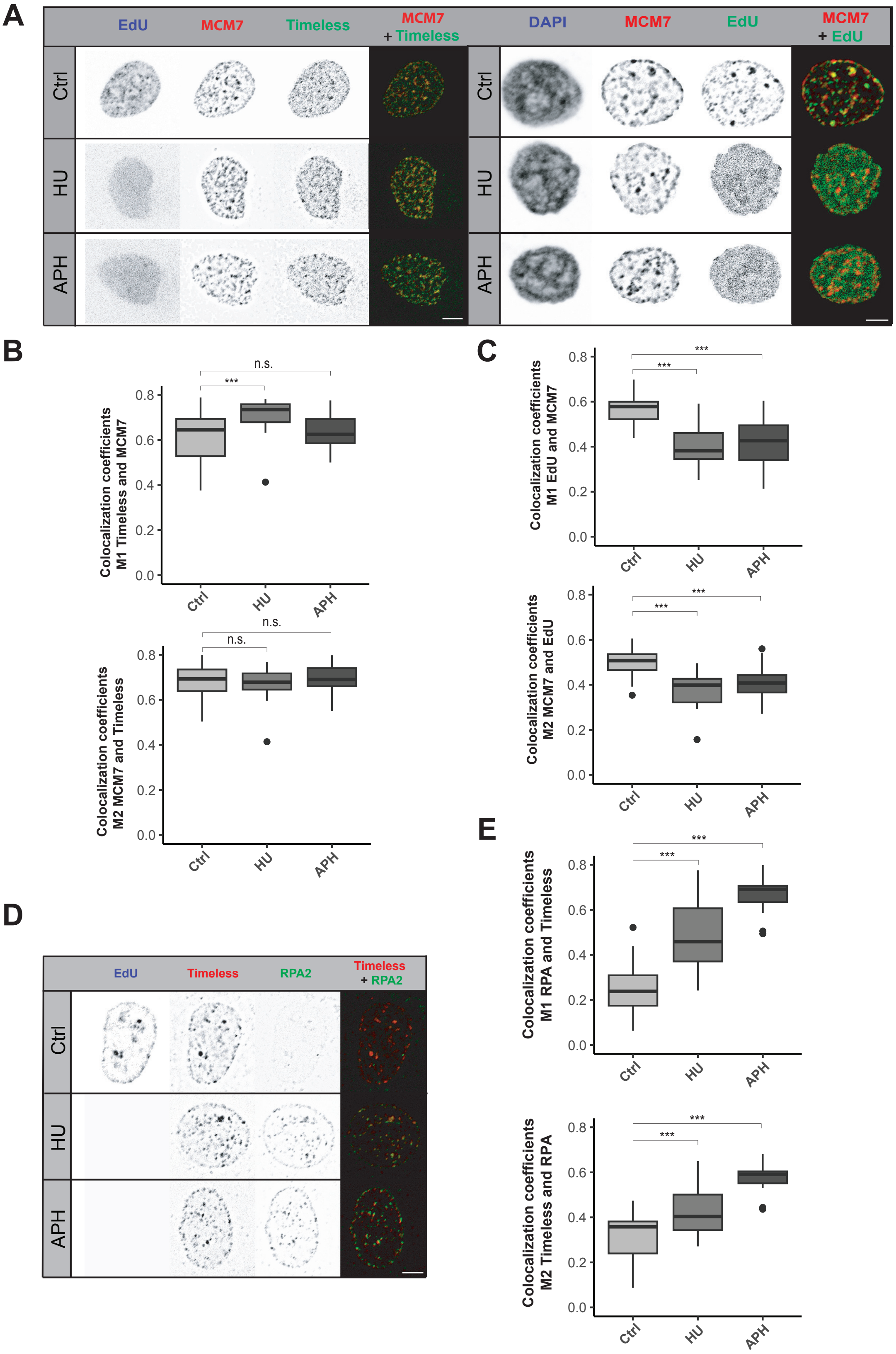
A
EdU
MCM7
Timeless
MCM7 + Timeless
DAPI
MCM7
EdU
MCM7 + EdU
Ctrl
HU
APH
B
Colocalization coefficients M1 Timeless and MCM7
n.s.
***
Colocalization coefficients M2 MCM7 and Timeless
n.s.
n.s.
C
Colocalization coefficients M1 EdU and MCM7
***
***
Colocalization coefficients M2 MCM7 and EdU
***
***
D
EdU
Timeless
RPA2
Timeless + RPA2
Ctrl
HU
APH
E
Colocalization coefficients M1 RPA and Timeless
***
***
Colocalization coefficients M2 Timeless and RPA
***
***
0.0
0.2
0.4
0.6
0.8
Ctrl
HU
APH

Figure S10. MCM helicase associates with Timeless in normal and stress conditions.

(A) Representative images of Timeless, MCM7 and EdU detection in HeLa Kyoto cells under the specified conditions. The experimental setup is as indicated in Figure 2B. Scale bar: 5 µm. **(B)** Manders coefficient M1 indicating colocalization of Timeless and MCM7 signals (upper panel). Manders coefficient M2 indicating colocalization of MCM7 and Timeless signals (lower panel). N (M1, M2) = 32 cells (Ctrl); 24 cells (HU); 23 cells (APH). **(C)** Manders coefficient M1 indicating colocalization of MCM7 and EdU signals (upper panel). Manders coefficient M2 indicating colocalization of EdU and MCM7 signals (lower panel) N (M1, M2) = 22 cells (Ctrl); 21 cells (HU); 25 cells (APH). **(D)** Representative images of Timeless, RPA and EdU detection in HeLa Kyoto cells stably expressing GFP-RPA2 under specified conditions. The experimental setup as indicated in Figure 2B. Scale bar: 5 µm. **(E)** Manders coefficient M1 indicating colocalization of Timeless and RPA2 signals (upper panel). Manders coefficient M2 indicating colocalization of RPA2 and Timeless signals (lower panel). N (M1, M2) = 31 cells (Ctrl); 46 cells (HU); 25 cells (APH). The statistical significance is indicated as: n.s. = not significant ($p > 0.05$); * = $p \leq 0.05$; ** = $p \leq 0.01$; *** = $p \leq 0.001$.

SUPPLEMENTARY TABLES

Supplementary Table 1: Cell line characteristics.

Name	Species	Type	Genotype	Reference
HeLa Kyoto	Homo sapiens	human carcinoma	-	(Erfle et al., 2007)
HeLa EGFP-PCNA	Homo sapiens	human carcinoma	pFRT-EGFP-PCNA	(Chagin et al., 2016)
HeLa EGFP-RPA2	Homo sapiens	human carcinoma	pFRT-EGFP-RPA2	(Pabba et al., 2023)
HEK 293-EBNA	Homo sapiens	human embryonic kidney	-	Invitrogen; Paisley, UK

Supplementary Table 2: Plasmid characteristics.

Name	Fluorophore	Gene	Lab collection number	Species	Promoter	Reference
pEGFP-Timeless	GFP	Timeless	pc4729	Homo sapiens	CMV	this study
pEGFP-Tipin	GFP	Tipin	pc4732	Homo sapiens	CMV	this study

Supplementary Table 3: Primers.

Name	Sequence 5' -> 3'	Application	Reference
hTimeless_HindIII_F	TAAGAT AAGCTT CG ATGGACTTGCACATGATGAACTG	cloning	this study
hTimeless_XbaI_R	TAAGATAAGCTTTCAGTC ATCCTCATCATCCTCAATC	cloning	this study
hTipin_ HindIII_F	TAAGATAAGCTT CGATG CTAGAACCACAGGAGAATGGCG	cloning	this study
hTipin_ XbaI_R	AACATA TCTAGA TCATCTAGC TTCAGTAATATTTCTGGATGTAG	cloning	this study
CMV_F	CGCAAATGGGCGGTAGGCGTG	sequencing	this study
Timeless 500 F	CTGACCTTGATCAGGAGAAGAAG	sequencing	this study
Timeless 1500 F	CTTCGGGAGAAAGCTCAGCA	sequencing	this study
Timeless 3000 F	CAGGATGTGGTGGAAGCCAT	sequencing	this study

Supplementary Table 4. Primary and secondary antibody characteristics.

Reactivity	Host	Dilution	Application	Cat #	Company/ Reference
Anti-Timeless	Rabbit	1:100	IF, WB	ab109512	Abcam (Cambridge, UK)
Anti-Timeless AlexaFluor 488-conjugated	Rabbit	1:100	IF	ab218278	Abcam (Cambridge, UK)
Anti-Tipin	Rabbit	1:100	IF, WB	AB_2648488	Thermofisher Scientific (Epson, UK)
Anti-RFP (clone 5F8)	Rat	1:20	IF	-	(Rottach et al., 2008)
Anti-MCM7	Rabbit	1:100	IF	2056-1	Epitomics Inc. (Burlingame, US)
Anti-MCM7	Mouse	1:100	PLA	sc-9966	Santa Cruz
Anti-RPA70A	Mouse	undiluted	IF, PLA	-	(Kenny et al., 1990)
Anti-rabbit IgG AlexaFluor 488	Donkey	1:400	IF (fluorescent secondary)	A11034	Invitrogen (Darmstadt, Germany)
Anti-mouse IgG AlexaFluor 488	Goat	1:400	IF (fluorescent secondary)	A11029	Invitrogen (Darmstadt, Germany)
Anti-rabbit IgG (H+L) AlexaFluor 594	Goat	1:500	IF (fluorescent secondary)	111-585-144	Jackson (Ely, UK)
Anti-rat IgG AlexaFluor 594	Donkey	1:500	IF (fluorescent secondary)	712-585-153	Jackson (Ely, UK)
Rabbit IgG Chromatographically purified	Rabbit	1:100	IF	55944	Organon Teknika (Oss, Niederlands)

Anti-rabbit IgG HRP	Goat	1:4000	WB (HRP-conjugated secondary)	A-0545	Sigma-Aldrich Chemie GmbH (Merck)
Anti-rat IgG HRP	Goat	1:4000	WB (HRP-conjugated secondary)	112-035-068	Jackson ImmunoResearch Europe Ltd.

Supplementary Table 5. Statistic parameters.

Secondary antibodies specificity verification					
**Figure**	**Name**	**Mean**	**Median**	**N**	**p-value**
S1C	R (Anti-rat IgG AF594)	0.080	0.080	2	-
S1C	R (Anti-rabbit IgG AF594)	-0.1417	-0.1350	6	-
S1C	R (Anti-rabbit IgG AF488)	0.050	0.050	2	-
S1C	R (Anti-rabbit IgG AF594 + Timeless AF488 conj)	0.04333	0.04000	6	
S1C	R (Anti-MCM7 + Timeless AF488 conj)	0.04	0.04	1	-
S1C	R (mCherry-PCNA + Timeless)	0.3885	0.3900	59	-

Colocalization mCherry-PCNA and Timeless/Tipin					
**Figure**	**Name**	**Mean**	**Median**	**N**	**p-value**
1D	R (Timeless)	0.3885	0.3900	59	0.287
1D	R (Tipin)	0.3623	0.3550	30	
S3C	M1 (Timeless)	0.4214	0.4210	59	0.05732
S3C	M1 (Tipin)	0.3672	0.3655	30	
S3D	M2 (Timeless)	0.3645	0.3550	59	0.02047
S3D	M2 (Tipin)	0.4108	0.4075	30	

Colocalization mCherry-PCNA and Timeless					
**Figure**	**Name**	**Mean**	**Median**	**N**	**p-value**
2C	R (Ctrl)	0.4356	0.43	25	
2C	R (HU)	0.2718	0.24	22	0.02065
2C	R (APH)	0.1246	0.12	24	1.342e-13
S3C	M1 (Ctrl)	0.4683	0.4665	25	
S3C	M1 (HU)	0.2990	0.3040	22	2.887e-07
S3C	M1 (APH)	0.1997	0.1850	24	1.268e-11
S3D	M2 (Ctrl)	0.4639	0.4710	25	

S3D	M2 (HU)	0.2645	0.2710	22	3.269e-08
S3D	M2 (APH)	0.1928	0.22	24	1.007e-10

**Colocalization Timeless and EdU**					
**Figure**	**Name**	**Mean**	**Median**	**N**	**p-value**
2D	R (Ctrl)	0.5821	0.59	28	
2D	R (HU)	0.4404	0.47	27	6.556e-06
2D	R (APH)	0.5167	0.54	21	0.02503
S3E	M1 (Ctrl)	0.6941	0.6895	28	
S3E	M1 (HU)	0.5137	0.536	27	5.715e-07
S3E	M1 (APH)	0.5671	0.524	21	0.0001354
S3F	M2 (Ctrl)	0.5962	0.59	28	
S3F	M2 (HU)	0.484	0.52	27	0.0004197
S3F	M2 (APH)	0.5275	0.527	21	0.0162

**Colocalization MCM7 and Timeless**					
**Figure**	**Name**	**Mean**	**Median**	**N**	**p-value**
3A	R (Ctrl)	0.5938	0.615	32	
3A	R (HU)	0.6083	0.63	24	0.7459
3A	R (APH)	0.59	0.59	23	0.3257
S3B	M1 (Ctrl)	0.6169	0.6455	32	
S3B	M1 (HU)	0.7211	0.7435	24	0.0005229
S3B	M1 (APH)	0.6422	0.625	23	0.3449
S3B	M2 (Ctrl)	0.6875	0.703	32	
S3B	M2 (HU)	0.672	0.6785	24	0.3164
S3B	M2 (APH)	0.6984	0.695	23	0.6147

**Colocalization MCM7 and EdU**					
**Figure**	**Name**	**Mean**	**Median**	**N**	**p-value**
3B	R (Ctrl)	0.4455	0.4550	22	
3B	R (HU)	0.319	0.330	21	8.395e-06
3B	R (APH)	0.34	0.33	25	0.0001295
S3C	M1 (Ctrl)	0.5670	0.5785	22	
S3C	M1 (HU)	0.3937	0.382	21	1.951e-08
S3C	M1 (APH)	0.4232	0.4270	25	1.481e-06
S3C	M2 (Ctrl)	0.4997	0.5075	22	
S3C	M2 (HU)	0.3763	0.399	21	7.607e-07
S3C	M2 (APH)	0.4142	0.408	25	6.104e-05

Colocalization EGFP-RPA and Timeless					
**Figure**	**Name**	**Mean**	**Median**	**N**	**p-value**
3C	R (Ctrl)	0.2103	0.19	31	
3C	R (HU)	0.4233	0.385	46	2.157e-08
3C	R (APH)	0.5196	0.52	25	< 2.2e-16
S3D	M1 (Ctrl)	0.2449	0.238	31	
S3D	M1 (HU)	0.4883	0.459	46	1.803e-12
S3D	M1 (APH)	0.6745	0.696	25	< 2.2e-16
S3D	M2 (Ctrl)	0.3184	0.358	31	
S3D	M2 (HU)	0.4259	0.404	46	0.0003468
S3D	M2 (APH)	0.5791	0.591	25	2.736e-10

Replication factor levels in replication					
**Figure**	**Name**	**Mean** [Intensity]	**Median** [Intensity]	**N**	**p-value** (S vs NS) (HU/APH vs Ctrl)
1C	Timeless S (not preextracted)	3305	3145	694	
1C	Timeless NS (not preextracted)	3148	2995	1254	6.822e-05
1C	Tipin S (not preextracted)	3568	3250	304	
1C	Tipin NS (not preextracted)	3816	3434	540	0.001376
1D	Timeless S (preextracted)	5164	5210	1460	
1D	Timeless NS (preextracted)	3490	3272	2745	< 2.2e-16
1D	Tipin S (preextracted)	8377	8232	961	
1D	Tipin NS (preextracted)	10384	10422	768	< 2.2e-16
2C	Timeless Ctrl	4913	4862	1109	
2C	Timeless HU	4941	4907	945	0.4472
2C	Timeless APH	5022	4978	1475	0.000251

Figure	Name	Mean<br>[Intensity Sum]	Median<br>[Intensity Sum]	N	p-value<br>(vs Ctrl)
S8A	PCNA Ctrl (not preextracted)	965472	945146	250	
S8A	PCNA HU (not preextracted)	918615	895196	578	0.1234
S8A	PCNA APH (not preextracted)	1255899	1247640	396	< 2.2e-16
S8A	RPA Ctrl (not preextracted)	664494	469530	380	
S8A	RPA HU (not preextracted)	697004	368054	996	0.9088
S8A	RPA APH (not preextracted)	1109999	977846	667	< 2.2e-16
S8A	MCM7 Ctrl (not preextracted)	1453718	1464913	783	
S8A	MCM7 HU (not preextracted)	1397643	1411135	705	0.001786
S8A	MCM7 APH (not preextracted)	1688397	1698840	371	< 2.2e-16
S8B	EdU Ctrl	883657	842641	1109	
S8B	EdU HU	756894	689134	945	7.761e-14
S8B	EdU APH	688007	666720	1475	< 2.2e-16
S8C	PCNA Ctrl (preextracted)	1008134	918273	539	
S8C	PCNA HU (preextracted)	853695	907707	295	0.007862
S8C	PCNA APH (preextracted)	777367	762579	254	0.00347
S8C	RPA Ctrl (preextracted)	356183	178488	847	
S8C	RPA HU (preextracted)	491744	309723	1383	< 2.2e-16
S8C	RPA APH (preextracted)	647624	337133	751	< 2.2e-16

PLA assay					
Figure	Name	Mean	Median	N	p-value
PLA controls (with one primary antibody)					
S2C	S phase (anti-MCM7)	0.09524	0	105	-
S2C	No S phase (anti-MCM7)	0.03754	0	293	-
S2C	S phase (anti-PCNA)	1.021	1	95	-
S2C	No S phase (anti-PCNA)	0.2646	0	223	-
S2C	S phase (anti-RPA)	1.129	1	139	-
S2C	No S phase (anti-RPA)	0.171	0	193	-
S2C	S phase (anti-Timeless)	0.1818	0	88	-
S2C	No S phase (anti-Timeless)	0.02326	0	215	-
PCNA Timeless					
2F	S phase (Ctrl)	20.060	19.000	330	
2F	S phase (HU)	4.290	3.000	293	< 2.2e-16
2F	S phase (APH)	1.708	1.000	315	< 2.2e-16
2F	No S phase (Ctrl)	2.538	1.000	377	< 2.2e-16
2F	No S phase (HU)	0.535	0.000	413	< 2.2e-16
2F	No S phase (APH)	0.402	0.000	757	< 2.2e-16
RPA Timeless					
3E	S phase (Ctrl)	1.52	1.00	327	
3E	S phase (HU)	4.431	4.000	211	< 2.2e-16
3E	S phase (APH)	9.239	8.000	355	< 2.2e-16
3E	No S phase (Ctrl)	0.452	0.000	325	< 2.2e-16
3E	No S phase (HU)	0.939	1.000	132	5.596e-08
3E	No S phase (APH)	1.269	1.000	201	0.001526
MCM7 Timeless					
3F	S phase (Ctrl)	5.323	5.000	229	
3F	S phase (HU)	6.000	4.000	272	0.644
3F	S phase (APH)	5.633	4.000	311	0.3369
3F	No S phase (Ctrl)	1.456	0.000	331	< 2.2e-16
3F	No S phase (HU)	2.352	1.000	341	< 2.2e-16
3F	No S phase (APH)	1.921	1.000	318	< 2.2e-16

SUPPLEMENTARY REFERENCES

Erfle, H., Neumann, B., Liebel, U., Rogers, P., Held, M., Walter, T., Ellenberg, J., and Pepperkok, R. (2007). Reverse transfection on cell arrays for high content screening microscopy. *Nat. Protoc.* 2, 392–399. doi:10.1038/nprot.2006.483.

Kenny, M. K., Schlegel, U., Furneaux, H., and Hurwitz, J. (1990). The role of human single-stranded DNA binding protein and its individual subunits in simian virus 40 DNA replication. *J. Biol. Chem.* 265, 7693–7700. doi:10.1016/S0021-9258(19)39170-7.

Rottach, A., Kremmer, E., Nowak, D., Leonhardt, H., and Cardoso, M. C. (2008). Generation and characterization of a rat monoclonal antibody specific for multiple red fluorescent proteins. *Hybridoma (Larchmt)* 27, 337–343. doi:10.1089/hyb.2008.0031.
